# The N-terminal disordered region of ChsB regulates its efficient transport to the hyphal apical surface in *Aspergillus nidulans*

**DOI:** 10.1007/s00294-023-01267-1

**Published:** 2023-04-18

**Authors:** Jingyun Jin, Ryo Iwama, Hiroyuki Horiuchi

**Affiliations:** 1 Department of Biotechnology, The University of Tokyo, Yayoi 1-1-1, Bunkyo-ku, Tokyo, 113-8657 Japan; 2 Collaborative Research Institute for Innovative Microbiology, The University of Tokyo, Yayoi 1-1-1, Bunkyo-ku, Tokyo, 113-8657 Japan; 3 Present Address: Peking University Institute of Advanced Agricultural Sciences, Shandong Laboratory of Advanced Agricultural Sciences in Weifang, Shandong, 261325 China

**Keywords:** *Aspergillus nidulans*, Chitin synthase, ChsB, Phosphorylation, Intrinsically disordered region

## Abstract

In fungi, the cell wall plays a crucial role in morphogenesis and response to stress from the external environment. Chitin is one of the main cell wall components in many filamentous fungi. In *Aspergillus nidulans*, a class III chitin synthase ChsB plays a pivotal role in hyphal extension and morphogenesis. However, little is known about post-translational modifications of ChsB and their functional impacts. In this study, we showed that ChsB is phosphorylated in vivo. We characterized strains that produce ChsB using stepwise truncations of its N-terminal disordered region or deletions of some residues in that region and demonstrated its involvement in ChsB abundance on the hyphal apical surface and in hyphal tip localization. Furthermore, we showed that some deletions in this region affected the phosphorylation states of ChsB, raising the possibility that these states are important for the localization of ChsB to the hyphal surface and the growth of *A. nidulans*. Our findings indicate that ChsB transport is regulated by its N-terminal disordered region.

**Supplementary Information:**

The online version contains supplementary material available at 10.1007/s00294-023-01267-1.

## Introduction

The fungal cell wall is one of the basic fungal cellular structures and plays a crucial role in fungal morphogenesis and response to stress from the external environment. The cell wall is also involved in fungal pathogenicity because it facilitates invasion into host tissues by protecting and hiding fungal cells under adverse conditions (Köhler et al. 2017). The fungal cell wall is primarily composed of chitin, glucan, and mannan (Free 2013; Gow et al. 2017). Chitin is synthesized from uridine diphosphate *N*-acetylglucosamine by chitin synthases, which imparts firmness to the cell wall by forming hydrogen bonds between chitin chains (Merzendorfer 2011). Various chitin synthases have been identified in different fungi and have been categorized into three divisions and seven classes according to their domain organizations (Horiuchi 2009; Rogg et al. 2012; Fajardo-Somera et al. 2015). Division 1 includes classes I, II, and III chitin synthases; Division 2 includes classes VI, V, and VII chitin synthases; and Division 3 includes class VI chitin synthases. The yeast *Saccharomyces cerevisiae* has three chitin synthases, namely, Chs1, Chs2, and Chs3, which are class I, II, and IV chitin synthases, respectively. Chs2 forms primary septa between mother and daughter cells, whereas Chs3 mainly forms the chitin in the cell wall and bud neck. Chs1 functions as a repair enzyme at the septation sites. The pathogenic dimorphic yeast *Candida albicans* has four chitin synthases, namely, *Ca*Chs1, *Ca*Chs2, *Ca*Chs3, and *Ca*Chs8, which belong to classes II, I, IV, and I, respectively. Transcriptome analyses of *S. cerevisiae* and *C. albicans* have revealed that the genes encoding chitin synthases are differentially expressed during the cell cycle and developmental stages (Chen-Wu et al. 1992; Pammer et al. 1992; Sudoh et al. 1993; Munro et al. 1998; Spellman et al. 1998; Côte et al. 2009). However, chitin synthase activities are not always correlated with gene expression levels (Choi et al. 1994) and can change under various conditions. In *S. cerevisiae*, the mutation of a β-1,3 glucan synthase increased the activities of Chs1 and Chs3 (García-Rodriguez et al. 2000). In *C. albicans*, the addition of a high concentration of calcium increased the activities of *Ca*Chs2 and *Ca*Chs8 (Munro et al. 2007). These results suggest that internal and/or external factors induce the post-translational modification and the regulation of chitin synthases, thereby regulating their activities.

Phosphorylation is one of the most investigated post-translational modifications of chitin synthases. In *S. cerevisiae*, Chs3 phosphorylation is dependent on Pkc1 activity. The activation of Pkc1 via heat shock is sufficient for Chs3 redistribution from the ER (Valdivia and Schekman 2003). Phosphoproteome analysis via mass spectrometry revealed that the N-terminal region of Chs3 contains some phosphorylation sites (Li et al. 2007; Albuquerque et al. 2008; Swaney et al. 2013). In *S. cerevisiae,* Chs2 has 12 phosphorylation sites in its N-terminal region, all of which are important for its stability (Martínez-Rucobo et al. 2009). Notably, Chs2 dephosphorylation can stimulate in vivo interaction with Sec24, one of the COPII components (Jakobsen et al. 2013), and is required for ER export of Chs2 (Teh et al. 2009; Chin et al. 2012). Both the phosphorylation and dephosphorylation of Ser217 in Chs2 play important roles in cytokinesis (Oh et al. 2012). In *C. albicans*, while *Ca*Chs3 can be phosphorylated at Ser139, both phosphorylation and dephosphorylation are required for its proper localization and function (Lenardon et al. 2010). These findings imply that phosphorylation of the N-terminal region is involved in important regulatory steps for various yeast chitin synthases.

Eight chitin synthases have been identified in the filamentous fungus *Aspergillus nidulans*. While the genes encoding class III, V, VI, and VII chitin synthases are not present in the genomes of *S. cerevisiae, Schizosaccharomyces pombe*, and *C. albicans*, *A. nidulans* has chitin synthases, with one or two under all the abovementioned classes. The functions of the six chitin synthases have been extensively analyzed (Horiuchi 2009; Rogg et al. 2012; Fernandes et al. 2016). The deletion of a class III chitin synthase-encoding gene, *chsB*, causes severe growth retardation, and its colonies form highly branched hyphae with abnormal morphologies (Yanai et al. 1994; Borgia et al. 1996). This finding suggests that ChsB plays a key role in the hyphal extension and morphogenesis of *A. nidulans*. ChsB is a membrane protein composed of 916 amino acids, and its behavior in hyphae has been well characterized. ChsB is primarily localized to the Spitzenkörper (Spk), the surface of hyphal tips, and the forming septa (Fukuda et al. 2009; Takeshita et al. 2015). ChsB is transported in vesicles on the microtubules by kinesin-1, to the hyphal apex regions and accumulates at the Spk. Subsequently, ChsB is transported to the plasma membrane at the hyphal tips and released there. ChsB then diffuses into the membrane and is internalized at the subapical hyphal collars via endocytosis. The internalized ChsB is recycled to the hyphal tips (Takeshita et al. 2015; Hernández-González et al. 2018; Zhou et al. 2018; Jin et al. 2021). However, the mechanism and the organelle into whose membrane ChsB is inserted remain to be elucidated.

Although ChsB has been suggested to be modified post-translationally (Fukuda et al. 2009; Takeshita et al. 2015), little is known about its implications on the proper localization of ChsB as well as on the regulation of this modification.

In this study, we showed that ChsB is a phosphorylated protein in *A. nidulans*. We investigated the N-terminal region of ChsB, which may have an intrinsically disordered region, and evaluated its involvement in the phosphorylation and localization of ChsB. We constructed strains that produced ChsB via the stepwise truncations of the N-terminal regions or the deletions of some residues in that region. Furthermore, we analyzed the effects of the deletions on the phosphorylation level and hyphal tip localization of ChsB as well as on fungal growth. The results revealed that the N-terminal region of ChsB determines its phosphorylation state, which significantly affects its hyphal tip localization and the growth of *A. nidulans*.

## Materials and methods

### Strains, media, and transformations

The *A. nidulans* strains used in this study are listed in Table S1. The strains were grown in a minimal medium (MMG) or a complete medium (YG) (Fukuda et al. 2009; Jin et al. 2021). When using a solid medium, 1.5% (w/v) agar was added. MMG or YG medium was supplemented with 0.5 mg/ml pyridoxine, 1.12 mg/ml uracil, 2.44 mg/ml uridine when necessary, and the additives were indicated by initials in lowercase letters after the medium name. The transformations of *A. nidulans* and *Escherichia coli*, and the propagation of *E. coli* were conducted as described previously (Tsuizaki et al. 2013).

### Construction of strains, plasmids and DNA fragments

The primers used in this study are listed in Table S2.

The 3FLChB strain, in which 3xFLAG-tagged ChsB was endogenously produced under the *chsB* promoter, was constructed as follows: A 1.5-kb fragment was amplified from the total DNA of the GPChBP strain using primers ‘5′-YFP-chsB for’ and ‘3xFLAG-chsB-1 rev.’ An 8.1-kb fragment was amplified from the total DNA of the GPChBP strain using primers ‘3xFLAG-chsB-2 for’ and ‘3′-YFP-chsB rev.’ The fragments were fused via PCR using primers ‘5′-YFP-chsB for’ and ‘3′-YFP-chsB rev’ to yield a 9.6-kb fragment. The 9.6-kb fragment was transformed into the A1149 strain. The transformants in which the gene encoding 3xFLAG-tagged ChsB was introduced into the 5′-terminus of *chsB* were selected using Southern blot analysis and designated as 3FLChB (Fig. S2A). The 3FLChBP strain, in which the wild-type *pyrG* was introduced into the 3FLChB strain, was constructed as follows: A 1.9-kb fragment was amplified from the total DNA of the A26 strain using primers ‘pyrG ORF-1 kb for’ and ‘pyrG ORF rev.’ The 1.9-kb amplified fragment was transformed into the 3FLChB strain. The transformant was designated as 3FLChBP.

A plasmid, pegfp–chsB, was constructed as follows: A 9.0-kb fragment was amplified from the total DNA of GPChBP strain using primers ‘5′-9xHA-chsB for’ and ‘3′-9xHA-chsB rev.’ The amplified fragment and pBlueScript II SK( +) were digested with Hind III and ligated to yield pegf–chsB.

The GPChBPΔ1–20, GPChBPΔ1–40, GPChBPΔ1–60, GPChBPΔ1–80, or GPChBPΔ1–100 strain that produced mutant ChsB with the deletion of the 1st–20th, 1st–40th, 1st–60th, 1st–80th, or 1st–100th amino acids at the N-terminus, respectively, instead of wild-type ChsB was constructed as described subsequently. A 2.1-kb fragment was amplified from the total DNA of GPChBP strain using primers ‘egfp-chsB-F’ and ‘3′-egfp rev’. DNA fragments were amplified from pegfp–chsB using primers ‘egfp-chsB-Δ1-20–2-F’ and ‘egfp-chsB-R’ for GPChBPΔ1–20; ‘egfp-chsB-Δ1-40–2-F’ and ‘egfp-chsB-R’ for GPChBPΔ1–40; ‘egfp-chsB-Δ1-60–2-F’ and ‘egfp-chsB-R’ for GPChBPΔ1–60; ‘egfp-chsB-Δ1-80–2-F’ and ‘egfp-chsB-R’ for GPChBPΔ1–80; and ‘egfp-chsB-Δ1-100–2-F’ and ‘egfp-chsB-R’ for GPChBPΔ1–100. Each fragment was then fused with the 2.1-kb fragment. Each of the obtained DNA fragments was transformed into the A1149/pyrG-1 strain (Katayama et al. 2012). The transformants in which the wild-type *chsB* was replaced with the introduced fragment were selected using Southern blot analysis and designated as GPChBPΔ1–20, GPChBPΔ1–40, GPChBPΔ1–60, GPChBPΔ1–80, and GPChBPΔ1–100, corresponding to the deleted regions of the ChsB N-termini.

The GPChBPΔ1–115 or GPChBPΔ1–140 strain that produced the mutant ChsB with the deletion of the 1st–115th or 1st–140th amino acids at the N-terminus, respectively, instead of wild-type ChsB, was constructed as follows: An 11.5-kb or 11.4-kb fragment was amplified from pegfp–chsB using primers ‘egfp-chsB-Δ1-115-F’ and ‘3′-egfp rev’ for GPChBPΔ1–115 or ‘egfp-chsB-Δ1-140-F’ and ‘3′-egfp rev’ for GPChBPΔ1–140, respectively. The 11.5-kb or 11.4-kb fragment was self-ligated to yield pegfp–chsB-Δ1–115 or pegfp–chsB-Δ1–140, respectively. The pegfp–chsB-Δ1–115 or pegfp–chsB-Δ1–140 strain was digested with Hind III to yield an 8.6-kb or 8.5-kb fragment, respectively. Each fragment was transformed into the A1149/pyrG-1 strain. The transformants in which the wild-type *chsB* was replaced with the introduced fragment were selected using Southern blot analysis and designated as GPChBPΔ1–115 or GPChBPΔ1–140, corresponding to the deleted regions of the ChsB N-termini.

The GPChBPΔ21–40, GPChBPΔ41–60, GPChBPΔ61–80, GPChBPΔ81–100, GPChBPΔ101–115 and GPChBPΔ116–140 strains that produced mutant ChsBs with the deletion of 1–20, 21–40, 41–60, 61–80, 81–100, 101–115, and 116–140 amino acids at the N-terminus, respectively, instead of wild-type ChsB, were constructed as described subsequently. DNA fragments encoding the N-terminal regions of ChsB were amplified from the total DNA of GPChBP strain using primers ‘egfp-chsB-F’ and ‘egfp-chsBΔ21-40–1-R’ for GPChBPΔ21–40; ‘egfp-chsB-F’ and ‘egfp-chsBΔ41-60–1-R’ for GPChBPΔ41–60; ‘egfp-chsB-F’ and ‘egfp-chsBΔ61-80–1-R’ for GPChBPΔ61–80; ‘egfp-chsB-F’ and ‘egfp-chsBΔ81-100–1-R’ for GPChBPΔ81–100; ‘egfp-chsB-F’ and ‘egfp-chsBΔ101-115–1-R’ for GPChBPΔ101–115; and ‘egfp-chsB-F’ and ‘egfp-chsBΔ101-140–1-R’ for GPChBPΔ116–140. DNA fragments encoding the C-terminal regions of ChsB were amplified from pegfp–chsB using primers ‘egfp-chsB-Δ21-40–2-F’ and ‘egfp-chsB-R’ for GPChBPΔ21–40; ‘egfp-chsB-Δ41-60–2-F’ and ‘egfp-chsB-R’ for GPChBPΔ41–60; ‘egfp-chsB-Δ61-80–2-F’ and ‘egfp-chsB-R’ for GPChBPΔ61–80; ‘egfp-chsB-Δ81-100–2-F’ and ‘egfp-chsB-R’ for GPChBPΔ81–100; ‘egfp-chsB-Δ101-115–2-F’ and ‘egfp-chsB-R’ for GPChBPΔ101–115; and ‘egfp-chsB-Δ115-140–2-F’ and ‘egfp-chsB-R’ for GPChBPΔ116–140. Each of the N-terminal and C-terminal fragments was fused using fusion PCR with primers ‘egfp-chsB-F’ and ‘egfp-chsB-R.’ Each obtained fragment was transformed into the A1149/pyrG-1 strain. The transformants in which the wild-type *chsB* was replaced with the introduced fragment were selected via Southern blot analysis and designated as GPChBPΔ21–40, GPChBPΔ41–60, GPChBPΔ61–80, GPChBPΔ81–100, GPChBPΔ101–115 or GPChBPΔ116–140, corresponding to the deleted regions of the ChsB N-termini.

### Southern blot analysis

Genomic DNA of the transformed strains was digested with the restriction enzymes listed in Figure S2, and transferred to Nylon Membrane, positively charged (Roche, Mannheim, Germany). Probes were amplified using PCR DIG Labeling Mix^PLUS^ (Roche) according to the manufacturer’s instructions. The probes were detected using Anti-Digoxigenin-AP (Roche) and CDP-Star™ detection reagent (GE Healthcare, IL, USA) according to the manufacturer’s instructions.

### Preparation of cell lysate

Total cell lysates were extracted as previously described (Jin et al. 2021). To determine the phosphorylation level of ChsB, urea was added to the lysate to a final concentration of 6 M, and the lysate was incubated at 100 °C for 5 min before performing SDS–polyacrylamide gel electrophoresis.

### Immunoprecipitation

Protein G FG beads (0.1 mg; TAS8848N1173; Tamagawa Seiki, Nagano, Japan) were washed with phosphate-buffered saline (PBS) (0.14 M NaCl, 8 mM Na_2_HPO_4_, 2.7 mM KCl, 1.5 mM KH_2_PO_4_) twice. PBS (10 μl) and 5 μl monoclonal anti-FLAG antibody produced in rabbit (F7425; Merck Millipore, MA, USA) or anti-GFP (118144600001; Roche) were added to the beads and centrifuged at 1400 rpm for 30 min at room temperature. The beads were washed three times with wash buffer (1 mM ethylenediaminetetraacetic acid [EDTA], 10% glycerol [v/v], 10 mM 4-[2-hydroxyethyl]-1-piperazineethanesulfonic acid sodium hydroxide [HEPES–NaOH; pH 7.9], and 50 mM KCl). Wash buffer (20 μl) and 200 μl KCl buffer (0.4 mM CaCl_2_, 0.4 mM EDTA and 20% glycerol [v/v], 40 mM HEPES–NaOH [pH 7.9], 300 mM KCl, 2 mM MgCl_2_, 0.2% NP-40 [w/v] and 0.4 mM phenylmethylsulfonyl fluoride) were added to the beads with agitation. The beads were magnetically separated after spin-down, and the supernatant was removed. The beads were washed twice with KCl buffer. The cell lysate was diluted to 1 mg/ml with KCl buffer, and 200 μl of the diluted lysate was added to the beads with agitation. After agitation at 4 °C for 2 h, the beads were washed three times with KCl buffer. 0.1 M Glycine–HCl buffer (28 μl; pH 2.5) was added to the beads with agitation, after which the beads were placed on ice for 5 min. After magnetic separation, the supernatant was collected and 2 μl of Tris–HCl buffer (pH 9.0) was added to obtain an immunoprecipitation fraction.

To purify EGFP-fused ChsBs, 500 µl of total cell lysate (protein concentration: 5 mg/ml) was added to 12.5 µl of GFP-Trap Magnetic Agarose (Proteintech Group, IL, USA). Bead equilibration, protein binding, washing, and elution with acidic elution buffer were conducted as described in the manufacturer’s documentation. The elution buffer was 50 µl, and the elution was performed twice.

### Phosphatase treatment

To dephosphorylate proteins, 3 μl of 10 × alkaline phosphatase buffer (Takara Bio, Shiga, Japan), 25 μl of calf intestine alkaline phosphatase buffer (CIAP; 1 mM MgCl_2_ and 50 mM Tris–HCl [pH 7.5]) and 3 μl of CIAP (Takara Bio) were added to 2 μl of the immunoprecipitation fraction and incubated at 37 °C for 2 h. Phosphatase inhibitor cocktail 1 (1 μl, Merck Millipore) and phosphatase inhibitor cocktail 2 (1 μl, Merck Millipore) were also added to the solutions.

To dephosphorylate EGFP-fused ChsBs, 10 µl of the elution fraction, 3 µl of 10 × Alkaline phosphatase buffer (Takara Bio), 2 μl of Alkaline Phosphatase (Calf intestine) (Takara Bio), and 15 µl of distilled water were mixed and incubated at 37 °C for 2 h.

### Measurement of colony area

The area of the colony was measured using Fiji (https://imagej.net/software/fiji/).

### Fluorescence microscopy and measurement of fluorescence intensity

Fluorescence microscopy and measurement of fluorescence intensity were conducted as previously described (Jin et al. 2021).

### CFW and CMAC staining

To observe the septa, the hyphae were treated with CFW solution (0.5 mg/mL fluorescent brightener 28 (F-3543; Sigma-Aldrich, MO, USA), 5% [w/v] potassium hydroxide, 10% [v/v] glycerol) for 1 min at room temperature. To visualize vacuoles, the hyphae were treated with 100 µM 7-amino-4-chloromethylcoumarin-L-alanyl-L-proline amide (CMAC-Ala-Pro; Y7531; Thermo Fisher Scientific, MA, USA) in MMG liquid medium for 30 min at room temperature in the dark. Fluorescence was observed using a U-MWU2 filter cube (Evident, Tokyo, Japan).

### Estimation of protein concentration

The protein concentration of the cell lysate was determined using a Protein Assay BCA kit (Fujifilm, Tokyo, Japan) according to the manufacturer’s instructions. Absorbance at 562 nm was measured using a UV-1900 spectrophotometer (Shimadzu, Kyoto, Japan).

### Immunoblot analysis

Immunoblot analysis was performed as described previously (Jin et al. 2021). Primary antibodies for β-tubulin, FLAG-tag, and GFP used in this study were monoclonal anti-β-tubulin antibody (clone: 10G10; Fujifilm), monoclonal anti-FLAG M2 antibody (F1804; Sigma-Aldrich), and monoclonal anti-GFP antibodies (clones: 7.1 and 13.1; 11814460001; Roche), respectively. Secondary antibodies were anti-mouse IgG and HRP-linked Antibody (7076S; Cell Signaling Technology, MA, USA). Chemiluminescence signals produced by ImmunoStar Zeta (Fujifilm) or SuperSignal West Atto Ultimate Sensitivity Substrate (Thermo Fisher Scientific) were detected using a CCD camera system (iBright FL1500 Imaging System; Thermo Fisher Scientific). Band intensities were quantified using Fiji Gel Analyzer.

### Statistical analysis

Statistical analysis was performed using R (https://www.r-project.org).

## Results

### ChsB is phosphorylated

**Fig. 1:**
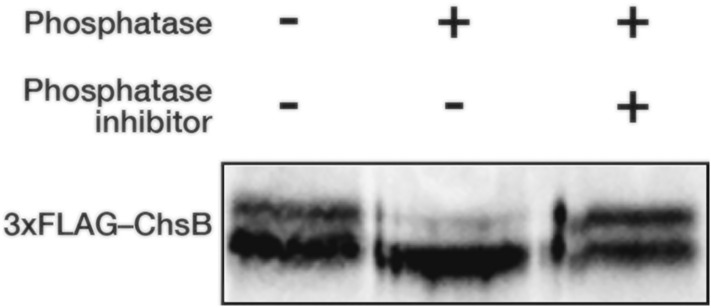
ChsB extracted from mycelia was phosphorylated. 2.0 × 10^7^ conidia of the 3xFLAG–ChsB producing strain were spread onto MMGpuu plate and incubated for 22 h at 30 °C, after which the mycelia were collected from approximately 1 cm margins of colonies. Total cell lysates were prepared from the mycelia and 3xFLAG–ChsB proteins were purified using magnetic beads conjugated with anti-FLAG antibodies. Purified proteins treated with or without a phosphatase and/or phosphatase inhibitors were subjected to immunoblot analysis using anti-FLAG antibodies

Immunoblot analysis of the cell lysate of the strain expressing 3xFLAG-tagged ChsB (3xFLAG–ChsB) revealed four bands (Fukuda et al. 2009; Takeshita et al. 2015). Treatment of the samples containing 3xFLAG–ChsB with 6 M urea before immunoblotting resulted in the convergence of the upper two bands to the lower two bands (Fig. 1 and data not shown). This finding suggests that ChsB contains a conformation that is relatively resistant to SDS denaturation but is disrupted after urea treatment. Moreover, as two bands were still detected after the treatment, 3xFLAG–ChsB might have been post-translationally modified in the cells. NetPhos-3.1 (https://services.healthtech.dtu.dk/service.php?NetPhos-3.1) (Blom et al. 1999, 2004), which predicts serine, threonine, and tyrosine phosphorylation sites in eukaryotic proteins, showed a number of potential phosphorylation sites on ChsB (those sites on the N-terminal region of ChsB are shown in Fig. S1E). To investigate whether ChsB is phosphorylated in the cells, we treated 3xFLAG–ChsB immunoprecipitated from the cell lysates of the 3FLChBP strain that produced 3xFLAG–ChsB with a phosphatase (Fig. 1). Phosphatase treatment dramatically reduced the upper band, whereas simultaneous treatment with the phosphatase and phosphatase inhibitor cocktails hardly reduced it. These results indicate that a portion of ChsB is phosphorylated in *A. nidulans*. Furthermore, the phosphatase treatment seemed to slightly increase the electrophoretic mobility of the lower band. Thus, ChsB is also possibly phosphorylated in the lower band.

### ChsB is predicted to have an intrinsically disordered region at its N-terminus

Recently, the overall structure of *Ca*Chs2, except for its N-terminal region (approximately 1–120 amino acids), has been examined using cryogenic electron microscopy (cryo-EM) (Ren et al. 2022). More recently, the structure of chitin synthase in the soybean pathogenic oomycete fungus *Phytophthora sojae* (*Ps*Chs1) was also determined using cryo-EM and was found to be similar to that of *Ca*Chs2 (Chen et al. 2022). These chitin synthases belong to the same division as ChsB. *Ca*Chs2 is composed of an N-terminal cytoplasmic glycosyltransferase domain, an interfacial domain, and six C-terminal transmembrane domains. However, the full-length protein structure of ChsB has not been described. Using the full-length amino acid sequence of ChsB (916 amino acids), the predicted structure of ChsB was obtained using AlphaFold2 (Jumper et al. 2021). Most of the C-terminal sequences were predicted with very high confidence (Fig. S1A). The overall predicted structure of ChsB was similar to that of *Ca*Chs2. Both structures included Chitin_synth_1N (PF08407), Chitin_synth_1 (PF01644), and transmembrane domains. In contrast, the model confidence for the N-terminal region (approximately 1–140 amino acids) was very low (Fig. S1B, and S1C). PrDOS (https://prdos.hgc.jp/cgi-bin/top.cgi) (Ishida and Kinoshita 2007), which predicts intrinsically disordered protein regions, indicated with a high probability that the N-terminal region of ChsB is an intrinsically disordered region (Fig. S1D). In general, when disordered proteins are post-translationally modified, the binding affinity of these proteins to their receptors is greatly altered (Dunker et al. 2001; Wright and Dyson 2015; Uversky 2019). In addition, the N-terminal disordered region of ChsB has many phosphorylatable residues, such as serine, threonine, and tyrosine (Fig. S1E). Thus, we hypothesized that the phosphorylation state in this region affects the dynamics and properties of ChsB.

### N-terminal disordered region of ChsB regulates its abundance at the hyphal tip

**Fig. 2:**
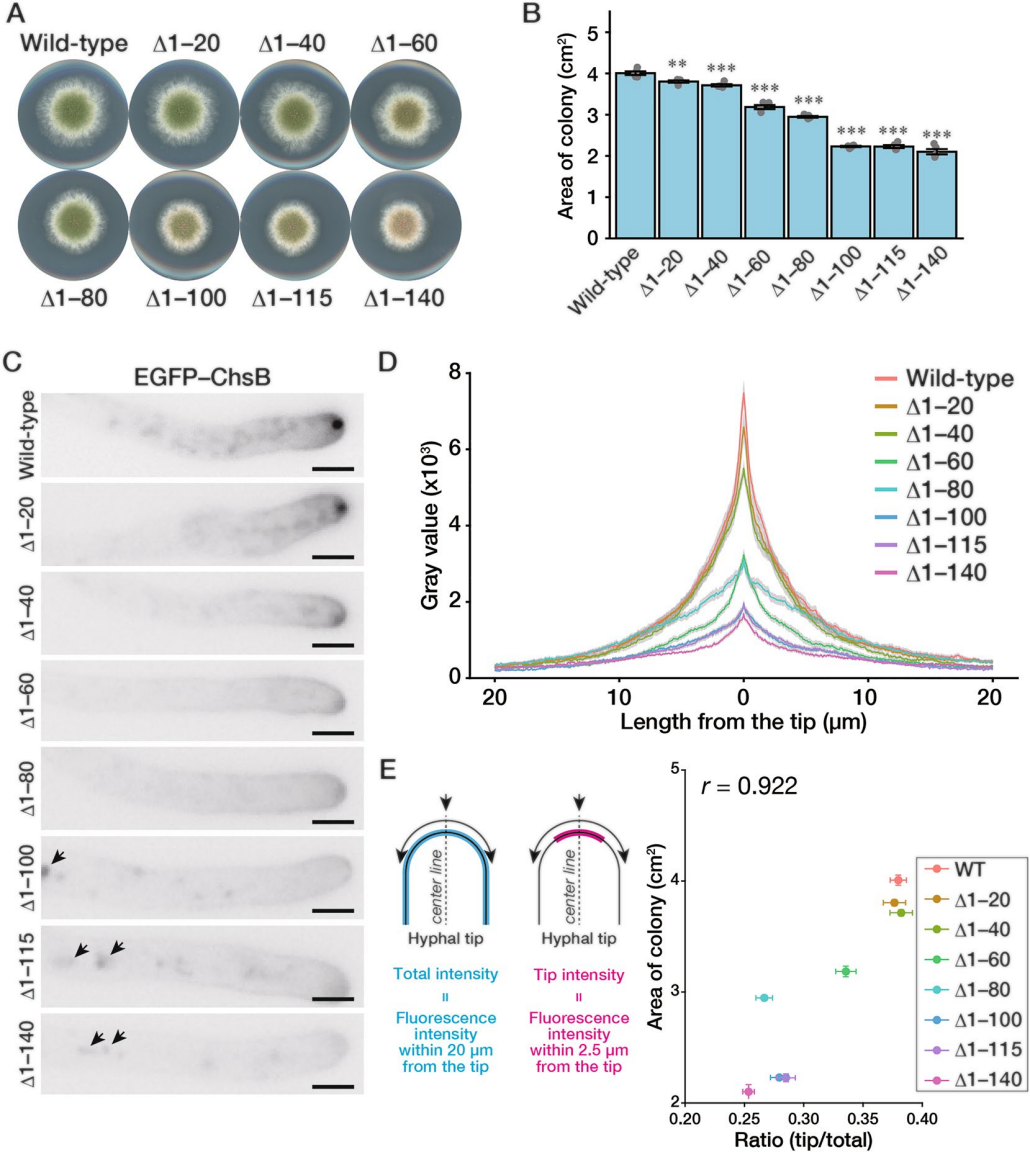
Truncation of the N-terminal disordered region perturbs ChsB localization to the hyphal apical surface. **A** 1.0 × 10^3^ conidia of the GPChBP (Wild-type), GPChBΔ1–20 (Δ1–20), GPChBΔ1–40 (Δ1–40), GPChBΔ1–60 (Δ1–60), GPChBΔ1–80 (Δ1–80), GPChBΔ1–100 (Δ1–100), GPChBΔ1–115 (Δ1–115), and GPChBΔ1–140 (Δ1–140) strains were inoculated onto MMGpuu plates and incubated for 72 h at 30 °C. **B** Colony areas of the strains described in (**A**) were measured. Bars indicate the mean of five independent experiments, and dots indicate raw data. Error bars represent S.E. Significant differences compared to the wild-type are indicated as asterisks (**P < 0.01; ***P < 0.001; Dunnett’s test). **C** 5.0 × 10^4^ conidia of the strains described in (**A**) were inoculated onto MMGpuu plates and incubated for 22 h at 30 °C, after which hyphae were observed using a fluorescence microscope. Arrows, abnormal intracellular foci. Bars: 5 μm. **D** The fluorescence intensities in (**C**) along their hyphal surfaces were measured. Lines indicate the average of the intensities, and gray regions indicate S.E. The number of measured hyphae is 24 (Wild-type), 24 (Δ1–20), 23 (Δ1–40), 21 (Δ1–60), 21 (Δ1–80), 24 (Δ1–100), 25 (Δ1–115), and 23 (Δ1–140). **E** The fluorescence intensities in (**D**) within 20 µm (total intensity) or 2.5 µm (tip intensity) from the hyphal tips (0 μm) were calculated. Then the ratio of tip intensity to total intensity was calculated. The calculated ratio and colony area in (**B**) were plotted; dots indicate the mean and error bars represent S.E.

When the N-terminal disordered region was deleted, *A. nidulans* showed growth retardation (Fig. 2A, ∆1–140). To investigate the impact of the region on the localization of ChsB and the growth of *A. nidulans*, we constructed strains that produced N-terminally truncated ChsBs in which EGFP was fused to their N-termini to observe their localizations (Fig. S2), instead of the wild-type ChsB. Since previous studies exhibited that deletion of *chsB* led to severe growth retardation (Yanai et al. 1994; Borgia et al. 1996), the fusion of EGFP to the N-terminus is not expected to cause dysfunction of ChsB (Fig. 2A, Wild-type). Growth retardation became more severe in the order of ChsB^∆1–20^, ChsB^∆1–40^, ChsB^∆1–60^, ChsB^∆1–80^, and ChsB^∆1–100^ (Fig. 2A and B). In these deletion mutants, relatively more pronounced effects were observed when the amino acids 41–60 or 81–100 were deleted. In contrast, the mutants of ChsB^∆1–100^, ChsB^∆1–115^, and ChsB^∆1–140^ showed growth retardation very similar to each other (Fig. 2A and B). These results indicate that the 100 N-terminal amino acids are required for the full function of ChsB and that the effects of N-terminal deletions depend on the N-terminal length. Despite growth retardation, no significant differences were observed in the hyphal morphologies of any of the N-terminally truncated ChsB-producing strains compared with those of the wild-type strain (Fig. S3A).

Next, we observed the localization of the truncated ChsBs in the hyphae (Fig. 2C). Punctate intracellular localizations of ChsB were observed in the hyphae of the strains producing EGFP–ChsB^∆1–100^, EGFP–ChsB^∆1–115^, and EGFP–ChsB^∆1–140^ (Fig. 2C, arrows). These punctae were co-localized with foci observed on CMAC-staining (Fig. S4), which indicates that these EGFP–ChsBs were transported into the vacuoles. Conversely, small amounts of EGFP–ChsBs were transported to the vacuoles in the other ChsB mutants (Fig. S4, contrast enhancement in EGFP–ChsB). Subsequently, we quantified EGFP signals along the hyphal surface and found that the deletions of amino acids 1–60 and 1–100 decreased the ChsB abundance on the hyphal tip surfaces compared with the deletions of amino acids 1–40 and 1–80, respectively (Fig. 2D). Furthermore, we observed that the graphs were not sharp in the strains producing EGFP–ChsB^∆1–80^, EGFP–ChsB^∆1–100^, EGFP–ChsB^∆1–115^, and EGFP–ChsB^∆1–140^. In these strains, the decreases in the relative signal intensities from the hyphal tips (0 μm) to the posterior parts (20 μm) were clearly smaller than those in the other strains (Fig. S5A). As the EGFP–ChsB signals in the strains defective in the endocytosis of ChsB showed similar patterns (Jin et al. 2021), these results suggest that the ChsB mutants with 80 or more amino acid deletions are not efficiently internalized at the subapical collars via endocytosis. We calculated the ratio of tip intensity to the total intensity and compared the ratio with the colony area (Fig. 2E). A strong correlation was found between these values (*r* = 0.922, Pearson’s correlation coefficient). Importantly, the EGFP–ChsB^∆1–80^-producing strain showed a relatively fast growth despite its low tip/total ratio (Fig. 2E). We assumed that the total ChsB abundance on the hyphal surface also affected the growth speed. To investigate this assumption, we categorized the mutant strains into two groups based on the internalization ratio of ChsB (Group 1: EGFP–ChsB, EGFP–ChsB^∆1–20^, EGFP–ChsB^∆1–40^, and EGFP–ChsB^∆1–60^; Group 2: EGFP–ChsB^∆1–80^, EGFP–ChsB^∆1–100^, EGFP–ChsB^∆1–115^, and EGFP–ChsB^∆1–140^) and plotted the total intensity on the hyphal surface and the colony area (Fig. S5B). Consistent with our assumption, a significant linear relationship was observed in each group (*R*^*2*^ = 0.989 and *R*^*2*^ = 0.997, respectively). These results imply that both the total ChsB abundance on the hyphal apical surface and the hyphal tip localization of ChsB are crucial for relatively rapid hyphal elongation. Furthermore, the results suggest that the N-terminal disordered region of ChsB is involved in regulating its properties.

As ChsB also localizes to forming septa and abnormal septal morphologies were observed in the *chsB* deletion mutant (Fukuda et al. 2009), we investigated septal shapes in the mutant strains (Fig. S6A). All the N-terminally truncated ChsB-producing strains showed septa with normal shapes. Furthermore, even EGFP–ChsB^∆1–140^, which had the least hyphal-tip localization rate, showed localization to forming septa along with the wild-type EGFP–ChsB (Fig. S6B), thereby suggesting that the N-terminal disorder region of ChsB is not essential for septum formation.

### N-terminal disordered region of ChsB controls its phosphorylation state

**Fig. 3:**
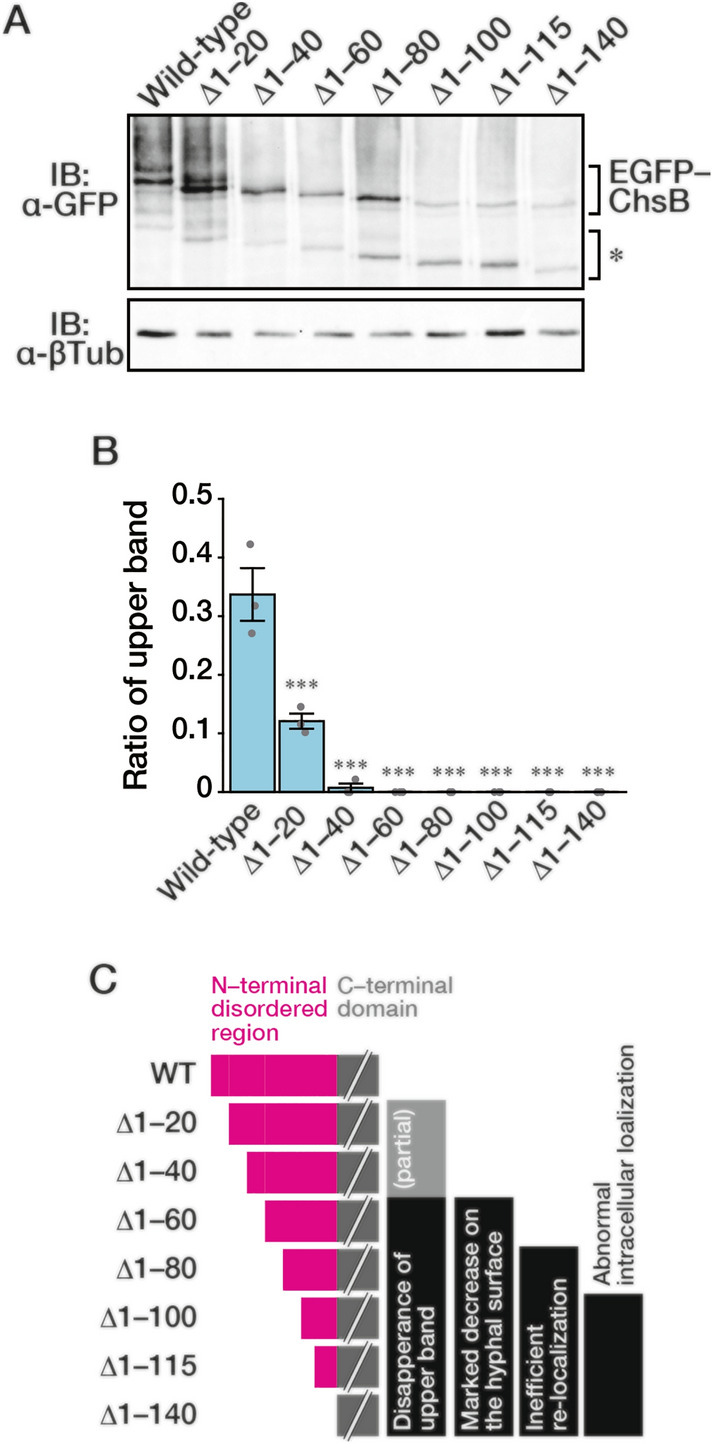
Truncation of the N-terminal disordered region diminishes the phosphorylated ChsB. **A** 2.0 × 10^7^ conidia of the GPChBP (Wild-type), GPChBΔ1–20 (Δ1–20), GPChBΔ1–40 (Δ1–40), GPChBΔ1–60 (Δ1–60), GPChBΔ1–80 (Δ1–80), GPChBΔ1–100 (Δ1–100), GPChBΔ1–115 (Δ1–115), and GPChBΔ1–140 (Δ1–140) strains were spread onto MMGpuu plates and incubated for 22 h at 30 °C, after which the mycelia were collected from approximately 1 cm margins of colonies. Total cell lysates were prepared from the mycelia and subjected to immunoblot analysis using anti-GFP (α-GFP) and anti-β-tubulin (α-βTub) antibodies. An asterisk indicates the bands derived from degraded products. **B** Signal intensities of the upper and lower bands of EGFP–ChsB were measured, and the ratio of the upper band intensity to total intensity was calculated. Bars indicate the mean of three independent experiments, and dots indicate raw data. Error bars represent S.E. Significant differences compared to the wild-type are indicated as asterisks (***P < 0.001; Dunnett’s test). **C** Summary of the results of the truncation mutants (see main text)

To investigate the effects of N-terminal truncation on ChsB modification, we performed immunoblot analysis of the total cell lysates extracted from these strains using an anti-GFP antibody (Fig. 3A). We found that the upper bands of EGFP–ChsB gradually disappeared as the N-terminus of ChsB shortened (Fig. 3B). Deletions of 60 or more amino acids at the N-terminus resulted in the almost complete disappearance of the upper band. These results suggest that the first 20 N-terminal amino acids of ChsB are important for the efficient phosphorylation of ChsB and that the first 60 N-terminal amino acids are required for producing the phosphorylation state in the upper band.

The results obtained from the microscopic observation and immunoblot analysis of the truncated ChsB are summarized in Fig. 3C. First, the N-terminal amino acids 1–100 are needed for the proper transportation of ChsB to the hyphal tips (Fig. 2C and D; Fig. 3C, Abnormal intracellular localization). Second, amino acids 1–80 are required for the efficient internalization of ChsB (Fig. S5A; Fig. 3C, Inefficient re-localization). Third, amino acids 1–60 are necessary for the phosphorylation state of ChsB corresponding to the upper bands in the immunoblot analysis (Fig. 3B; Fig. 3C, Disappearance of the upper band), and the same region is important for efficient ChsB recruitment to hyphal tips (Fig. 2D; Fig. 3C, Marked decrease on the hyphal surface). Furthermore, phosphorylation levels were also significantly reduced when amino acids 1–20 or 1–40 were deleted (Fig. 3B, C, (partial)). These results clearly show that the N-terminal disordered region of ChsB regulates various ChsB characteristics.

Immunoblot analysis revealed several bands below, which corresponded to the predicted size of each truncated EGFP–ChsB; these bands were considered to be degraded products (Fig. 3A, asterisk). The band of each primary degradation product was approximately 20–25 kDa smaller than that corresponding to the full length of each truncated EGFP-ChsB. Considering that EGFP was fused to the N-terminus, the degradation bands should be moieties that have lost at least two transmembrane regions at the C-termini in ChsBs. As the predicted structure of ChsB contains six transmembrane domains at the C-terminus (Fig. S1), these degradation products probably did not function properly, with only four transmembrane domains at their C-termini. As the punctate intracellular foci of EGFP–ChsB observed in some N-terminally truncated ChsB-producing strains were co-localized with the vacuoles (Fig. S4), these degradation products are possibly EGFP–ChsBs transported to and degraded in the vacuoles.

### Phosphorylation state of ChsB is involved in its recruitment to the hyphal apical surface

**Fig. 4:**
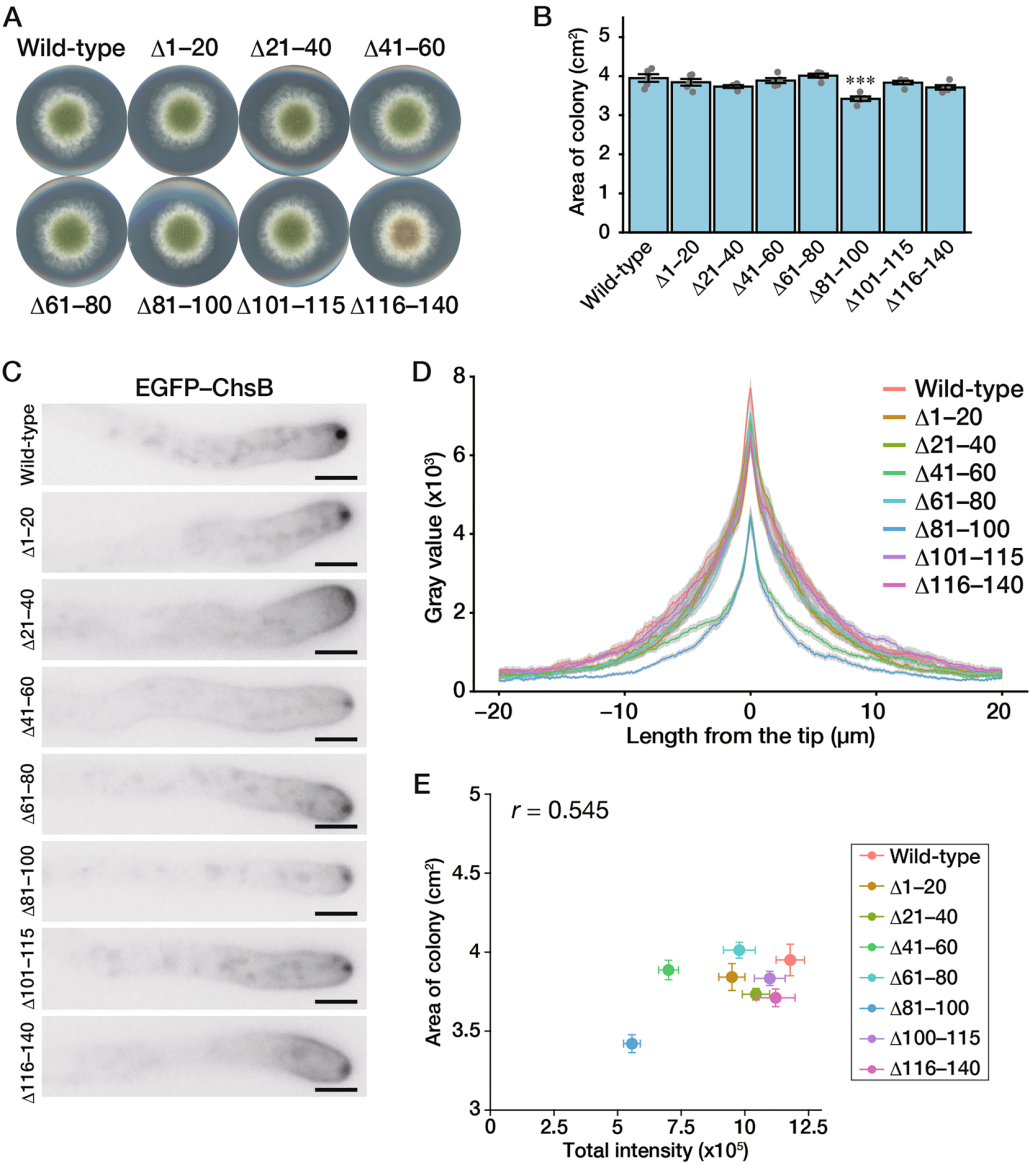
Deletion of amino acids 41–60 or 81–100 reduces ChsB localization at the hyphal apical surface. **A** 1.0 × 10^3^ conidia of the GPChBP (Wild-type), GPChBΔ1–20 (Δ1–20), GPChBΔ21–40 (Δ21–40), GPChBΔ41–60 (Δ41–60), GPChBΔ61–80 (Δ61–80), GPChBΔ81–100 (Δ81–100), GPChBΔ100–115 (Δ100–115), and GPChBΔ115–140 (Δ115–140) strains were inoculated onto MMGpuu plates and incubated for 72 h at 30 °C. **B** Colony areas of the strains described in (**A**) were measured. Bars indicate the mean of five independent experiments, and dots indicate raw data. Error bars represent S.E. Significant differences compared to the wild-type are indicated asterisks (***P < 0.001; Dunnett’s test). **C** 5.0 × 10^4^ conidia of the strains described in (**A**) were inoculated onto MMGpuu plates and incubated for 22 h at 30 °C, after which hyphae were observed using a fluorescence microscope. Bars: 5 μm. **D** The fluorescence intensities in (**C**) along their hyphal surfaces were measured. Lines indicate the average of the intensities, and gray regions indicate S.E. The number of measured hyphae is 23 (Wild-type), 26 (Δ1–20), 19 (Δ21–40), 23 (Δ41–60), 23 (Δ61–80), 25 (Δ81–100), 23 (Δ101–115), and 23 (Δ116–140). **E** The fluorescence intensities in (**D**) within 20 µm from the hyphal tips (0 μm) were calculated, and then the intensity and colony area in (**B**) were plotted; dots indicate the mean and error bars represent S.E.

We examined whether some specific parts of the N-terminal disordered region were essential for the expression of ChsB characteristics. We constructed and characterized the strains that produce EGFP-tagged ChsB^∆1–20^, ChsB^∆21–40^, ChsB^∆41–60^, ChsB^∆61–80^, ChsB^∆81–100^, ChsB^∆101–115^, and ChsB^∆116–140^ (Fig. S2). When amino acids 81–100 were deleted, significant growth retardation was observed at 30 °C, whereas the other deletions did not cause growth retardation (Fig. 4A and B). In addition, none of the amino acid deletions caused significant changes in the hyphal morphologies (Fig. S3B). Subsequently, we observed the subcellular localization of the mutant ChsBs and quantified the EGFP signals along the hyphal surface (Fig. 4C, D, S7). Unlike EGFP–ChsBs with deletions of 100 or more amino acids, of which significant portions were transported to the vacuoles, all the EGFP-tagged ChsBs with deletions of 20 amino acids were slightly detected in the vacuoles (Fig. S7). The total fluorescence intensity was decreased in EGFP–ChsB^∆41–60^ and EGFP–ChsB^∆81–100^, but the other mutant ChsBs showed patterns similar to that of the wild type (Fig. 4D). In contrast, no significant changes were observed in the decline patterns of the relative signal intensities in these mutants (Fig. S8), suggesting that all the mutant ChsBs with deletions of 20 amino acids were properly internalized. As the total intensity on the hyphal surface and colony area were correlated (*r* = 0.545, Pearson’s correlation coefficient) (Fig. 4E), the growth retardation of the strains producing ChsB^∆81–100^ may be due to the decreased total ChsB abundance on the hyphal surface. Although the total fluorescence intensity was decreased in EGFP–ChsB^∆41–60^ (Fig. 4D and E), the strain producing it did not show growth retardation at 30 °C (Fig. 4A and B). However, the strain showed a significant growth delay under incubation at 37 °C (Fig. S9), which suggests that the total ChsB abundance on the hyphal surface is more important for growth at higher temperatures.

**Fig. 5:**
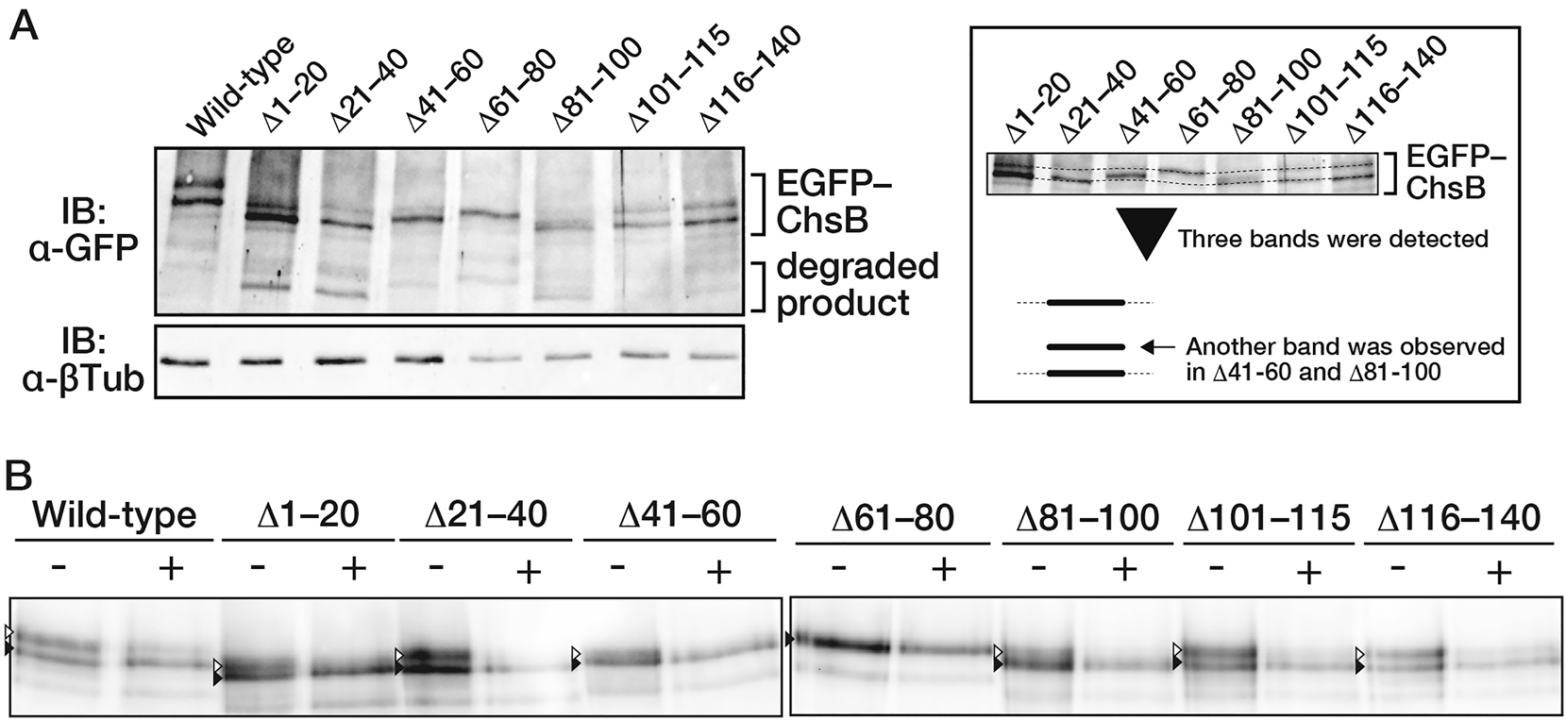
Amino acid deletions of the N-terminal region alter the phosphorylation state of ChsB. **A** (Left) 2.0 × 10^7^ conidia of the GPChBP (Wild-type), GPChBΔ1–20 (Δ1–20), GPChBΔ21–40 (Δ21–40), GPChBΔ41–60 (Δ41–60), GPChBΔ61–80 (Δ61–80), GPChBΔ81–100 (Δ81–100), GPChBΔ100–115 (Δ100–115), and GPChBΔ115–140 (Δ115–140) strains were spread onto MMGpuu plates and incubated for 22 h at 30 °C, after which the mycelia were collected from approximately 1 cm margins of colonies. Total cell lysates were prepared from the mycelia and subjected to immunoblot analysis using anti-GFP (α-GFP) and anti-β-tubulin (α-βTub) antibodies. (Right) The trimmed view around EGFP–ChsB. **B** EGFP–ChsBs were purified using GFP-trap from total cell lysates described in (**A**). Purified proteins treated with ( +) or without ( −) a phosphatase were subjected to immunoblot analysis using anti-GFP antibodies. White arrowheads indicate the detected bands that were decreased with the phosphatase treatment, while black arrowheads indicate the bands that were not changed with the phosphatase treatment. Under the phosphatase-treated condition, 10 µL of elution fraction was loaded; in the phosphatase-free condition, 30 µL of elution fraction was loaded

Immunoblot analysis of the total cell lysates of the mutant strains was performed (Fig. 5A). We found that in the EGFP–ChsB^∆41–60^- and EGFP–ChsB^∆81–100^-producing strains, another band was detected between the upper and lower bands (Fig. 5A, right box). We also found that the lower bands were almost undetectable in the EGFP–ChsB^∆41–60^- and EGFP–ChsB^∆81–100^-producing strains (Fig. 5A, right box). Subsequently, we purified the EGFP–ChsB moieties from all the mutant ChsB-producing strains and treated them with a phosphatase (Fig. 5B). Except for the EGFP–ChsB^∆61–80^ protein, phosphatase treatment reduced the amount of each upper band. This result suggests that these mutant ChsBs are phosphorylated in vivo. In ChsB^∆41–60^ and ChsB^∆81–100^, the ratios of the phosphorylated moieties to the non-phosphorylated ones were relatively low (Fig. 5B). Considering that the amino acid deletion mutants other than ChsB^∆41–60^ and ChsB^∆81–100^ showed total intensities similar to that of the wild-type ChsB on the hyphal surface (Fig. 4D), a certain amount of the phosphorylated moieties would be required for ChsB to be efficiently localized to the hyphal apical surface to the same extent as the wild-type ChsB. In the case of EGFP–ChsB^∆61–80^, the band was not shifted with the phosphatase treatment (Fig. 5B). However, the mobility of the band was similar to the upper bands observed in most of the strains. This suggests that EGFP–ChsB^∆61–80^ was modified (see “Discussion”).

**Fig. 6:**
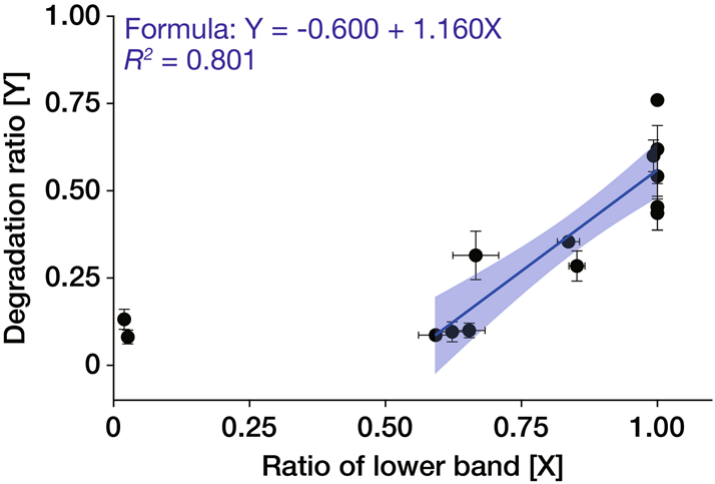
Ratio of the lower band of ChsB is correlated with its stability. Ratios of the amounts of degraded products to the total EGFP–ChsBs were calculated from immunoblot analysis in Fig. 3A and 5A. Then, the degradation ratios and the ratios of the lower bands of the total EGFP–ChsBs were plotted

Finally, we focused on the stability of ChsB. In the immunoblotting results shown in Figs. 3A and 5A, we plotted the ratio of the lower band and that of the bands derived from degradation products (Fig. 6). We observed a correlation between the two plotted ratios in the range where the ratio of the lower band was approximately > 0.5. These results suggest that unphosphorylated ChsB may be more susceptible to degradation than phosphorylated ChsB.

## Discussion

Disordered regions are widespread in eukaryotic proteins and are frequently the sites for post-translational modifications that alter the physical characteristics of the regions and play critical roles in signaling pathways (Wright and Dyson 2015). In the present study, we confirmed the influence of the N-terminal disordered region on the localization of ChsB and the growth of *A. nidulans*. Certain 20 amino acid deletions in the N-terminal disordered region affected the ChsB phosphorylation states, which were correlated with ChsB abundance on the hyphal surface. In addition, the growth retardation in the strain producing EGFP–ChsB^∆1–100^ was more severe than that in the strain producing EGFP–ChsB with any 20 amino acid deletion (Fig. 2B vs Fig. 4B). This result indicates that two or more parts in the ChsB N-terminal disordered region are required for the normal growth of *A. nidulans*.

In *S. cerevisiae*, Chs3 catalyzes the synthesis of approximately 90% of cellular chitin (Shaw et al. 1991), and has been studied as a model protein in membrane traffic studies (Feyder et al. 2015; Sánchez and Roncero 2022). The N-terminal cytosolic region of Chs3 regulates its oligomerization, and this assembly is important for proper Chs3 transport (Sacristan et al. 2013; Gohlke et al. 2017). Moreover, the N-terminal distinct regions of Chs3 play different roles in Chs3 transport to and from the plasma membrane (Weiskoff and Fromme 2014). In our experiments, the growth retardation of the strain producing EGFP–ChsB^∆1–60^ or EGFP–ChsB^∆1–100^ was more significant than that of the strain producing EGFP–ChsB^∆1–40^ or EGFP–ChsB^∆1–80^, respectively. Furthermore, the hyphal tip intensity of EGFP–ChsB^∆1–60^ or EGFP–ChsB^∆1–100^ was significantly decreased compared with that of EGFP–ChsB^∆1–40^ or EGFP–ChsB^∆1–80^, respectively (Fig. 2B and D). These results indicate that the regions between the N-terminal amino acids 41–60 and 81–100 are important for fungal growth and the localization of ChsB to the hyphal tips. This idea is supported by the fact that the deletion of amino acids 41–60 or 81–100 impeded fungal growth retardation and decreased the ChsB abundance on the hyphal apical surface, although growth retardation caused by the deletion of amino acids 41–60 was only observed at 37 °C (Fig. 4B, D, and S9). In the deletion mutant of the amino acids 41–60 or 81–100, the amounts of the phosphorylated moieties were slightly small (Fig. 5B). This finding suggests that the phosphorylation state of ChsB is critical for the localization of ChsB to the hyphal tips and the growth of *A. nidulans*. In this study, the mobility of EGFP–ChsB^∆61–80^ was not changed after a phosphatase treatment (Fig. 5B). This result suggests that EGFP–ChsB^∆61–80^ is not phosphorylated. Since the mobility of the band of EGFP–ChsB^∆61–80^ is relatively small compared with those of the other proteins, modifications other than phosphorylation may occur in EGFP–ChsB^∆61–80^. However, we cannot exclude the possibility that EGFP–ChsB^∆61–80^ is phosphorylated and that the phosphatase could not act on EGFP–ChsB^∆61–80^ due to changes in its local structural change or other factors. In the present study, we did not identify the specific ChsB phosphorylation sites. Many amino acid residues of the N-terminal regions of Chs2 and Chs3 are phosphorylated in *S. cerevisiae* (Li et al. 2007; Albuquerque et al. 2008; Martínez-Rucobo et al. 2009; Swaney et al. 2013), and Ser139 of *Ca*Chs3 is phosphorylated in *C. albicans* (Lenardon et al. 2010). The upper band was not detected in the truncation mutants of N-terminal 60 or more amino acids (Fig. 3A and B), which suggests that this region is either phosphorylated or required for phosphorylating other regions.

EGFP–ChsB^∆1–60^ was more efficiently internalized into the cell than EGFP–ChsB^∆1–80^ (Fig. 2D and S5A), which implies that the region between amino acids 61 and 80 is required for the efficient endocytosis of ChsB. Although the deletion mutant of amino acids 61–80 showed a similar pattern of intensity decline at the hyphal surface to that of the wild-type strain, other region(s) within the N-terminal 60 amino acids may also be involved in the efficient endocytosis of ChsB. In addition, when comparing EGFP–ChsB^∆1–80^ and EGFP–ChsB^∆1–100^, although no upper band was detected in either case, intracellular vacuolar localization was observed in the hyphae of EGFP–ChsB^∆1–100^ (Fig. 2C, D, and S4), which indicates that the length and/or some sections of the N-terminal disordered region have separate functions.

We previously showed that ChsB is efficiently endocytosed by the AP-2 complex (Jin et al. 2021). However, no canonical AP-2 complex recognition motifs, YxxΦ (Φ representing bulky hydrophobic amino acids) and [DE]xxxL[LI] (Ohno et al. 1995; Owen and Evans 1998; Doray et al. 2007; Kelly et al. 2008), were found in the N-terminal 100 amino acids of ChsB (data not shown). Further areas of interest would be whether the AP-2 complex binds to the N-terminal region and whether this region increases the binding affinity of the AP-2 complex to ChsB. Future research should also identify the proteins that interact with the N-terminal disordered region of ChsB to elucidate the mechanisms that regulate its localization.

Disordered regions exist in the N-termini of other class III chitin synthases, such as those of *Aspergillus fumigatus*, *Exophiala dermatitidis*, and *Neurospora crassa*, which belong to Ascomycota, and *Cryptococcus neoformans*, which belongs to Basidiomycota (Fig. S10A). Although amino acid identities in their sequences are low (Fig. S10B), sequence similarity is observed among *An*ChsB, *Af*ChsG, and *Ed*Chs3, which signifies that the roles of the disordered regions are conserved to some extent in class III chitin synthases of closely related species.

*A. nidulans* has eight chitin synthases, and these proteins are categorized into three divisions; ChsC, ChsA, ChsB, and ChsF belong to Division 1; ChsD, CsmA, and CsmB belong to Division 2; and ChsG belongs to Division 3. Except for CsmA and CsmB, both of which possess a myosin motor-like domain at their N-termini (Takeshita et al. 2002, 2006), the other six chitin synthases are expected to have a disordered region of approximately 150 amino acids at their N-termini (ChsA, ChsB, ChsC, ChsD, and ChsF) or the C-terminus (ChsG) as per the prediction on PrDOS (Ishida and Kinoshita 2007). The presence of these regions suggests that the localization of these chitin synthases may be regulated in a manner similar to that of ChsB.

## Supplementary Information

Below is the link to the electronic supplementary material.

Supplementary file1 ChsB is predicted to have an intrinsically disordered region at its N-terminus. **A**–**D** The predicted full-length structure of ChsB by AlphaFold2. A Side view of the structure. Red α-helices indicate predicted transmembrane domains. **B** The predicted disordered region at N-terminus is colored in magenta. **C** Top view of (**B**). **D** Amino acids sequences of ChsB were analyzed by the PrDOS server. **E** The first 140 amino acid sequences of ChsB are shown. Serine, threonine, and tyrosine residues are highlighted. Asterisks indicate predicted phosphorylation sites by NetPhos-3.1 (JPG 2522 KB)

Supplementary file2 Southern analyses of the strains constructed in this study. **A**, **B** Schemes of the southern analysis are depicted. **C**, **D** The results of the analysis. Black and white arrowheads indicate the postions at 5.4-kb and 9.0-kb bands, respectively. Panels **A** and **B** are the schemes of Panels **C** and **D**, respectively (JPG 1972 KB)

Supplementary file3 Truncations or the amino acid deletions of the N-terminal disordered region did not affect hyphal morphology. **A** Conidia of the GPChBP (Wild-type), GPChBΔ1–20 (Δ1–20), GPChBΔ1–40 (Δ1–40), GPChBΔ1–60 (Δ1–60), GPChBΔ1–80 (Δ1–80), GPChBΔ1–100 (Δ1–100), GPChBΔ1–115 (Δ1–115), and GPChBΔ1–140 (Δ1–140) strains were inoculated onto MMGpuu plates and incubated for 22 h at 30 °C, after which the hyphal morphologies were observed under a microscope. **B** Conidia of the GPChBP (Wild-type), GPChBΔ1–20 (Δ1–20), GPChBΔ21–40 (Δ21–40), GPChBΔ41–60 (Δ41–60), GPChBΔ61–80 (Δ61–80), GPChBΔ81–100 (Δ81–100), GPChBΔ100–115 (Δ100–115), and GPChBΔ115–140 (Δ115–140) strains were inoculated onto MMGpuu plates and incubated for 22 h at 30 °C, after which the hyphal morphologies were observed under a microscope. Bars: 5 µm (JPG 1657 KB)

Supplementary file4 Deletion of amino acids 1–100, 1-115, and 1-140 strongly alter ChsB localization to the vacuoles. Conidia of the GPChBP (Wild-type), GPChBΔ1–20 (Δ1–20), GPChBΔ1–40 (Δ1–40), GPChBΔ1–60 (Δ1–60), GPChBΔ1–80 (Δ1–80), GPChBΔ1–100 (Δ1–100), GPChBΔ1–115 (Δ1–115), and GPChBΔ1–140 (Δ1–140) strains were inoculated onto MMGpuu plates and incubated for 22 h at 30 °C, after which the hyphae were treated with CMAC-Ala-Pro and were observed under a fluorescence microscope. Bars: 3 µm (JPG 1817 KB)

Supplementary file5 Endocytosis of ChsB and total ChsB abundance on the hyphal surface are both involved in the hyphal elongation. **A** Using the data in Fig. 2B, relative values of the intensities were calculated concerning those at the hyphal tips. The intensities at the hyphal tips (0 μm) were set to 1. **B** The fluorescence intensities in Fig. 2D within 20 µm from the hyphal tips (0 μm) were calculated, and then the intensity and the area of the colony in Fig. 2B were plotted; dots indicate the mean and error bars represent S.E. Linear regression analysis was performed in each group (JPG 883 KB)

Supplementary file6 Truncations of the N-terminal disordered region did not affect septum formation. **A** Conidia of the GPChBP (Wild-type), GPChBΔ1–20 (Δ1–20), GPChBΔ1–40 (Δ1–40), GPChBΔ1–60 (Δ1–60), GPChBΔ1–80 (Δ1–80), GPChBΔ1–100 (Δ1–100), GPChBΔ1–115 (Δ1–115), and GPChBΔ1–140 (Δ1–140) strains were inoculated onto MMGpuu plates and incubated for 20 h at 30 °C, after which the hyphae were treated with CFW and were observed under a fluorescence microscope. **B** Conidia of the GPChBP and GPChBΔ1–140 strains were inoculated onto MMGpuu plates and incubated for 20 h at 30 °C, after which EGFP-ChsBs at forming septa were observed under a fluorescence microscope. Bars: 3 µm (JPG 1925 KB)

Supplementary file7 None of the 20-amino acid deletions of the N-terminal disordered region strengthened the vacuolar localization of ChsB. Conidia of the GPChBP (Wild-type), GPChBΔ1–20 (Δ1–20), GPChBΔ21–40 (Δ21–40), GPChBΔ41–60 (Δ41–60), GPChBΔ61–80 (Δ61–80), GPChBΔ81–100 (Δ81–100), GPChBΔ100–115 (Δ100–115), and GPChBΔ115–140 (Δ115–140) strains were inoculated onto MMGpuu plates and incubated for 20 h at 30 °C, after which the hyphae were treated with CMAC-Ala-Pro and were observed under a fluorescence microscope. Bars: 3 µm (JPG 1859 KB)

Supplementary file8 None of the 20-amino acid deletions of ChsB affected the internalization rate of ChsB. Using the data in Fig. 4B, relative values of the intensities were calculated concerning those at the hyphal tips. The intensities at the hyphal tips (0 μm) were set to 1 (JPG 974 KB)

Supplementary file9 Deletion of amino acids 41–60 retarded the growth at a high temperature. Colony areas of the strains described below were measured. Bars indicate the mean of five independent experiments, and dots indicate raw data. Error bars represent S.E. Significant differences compared to the wild-type are indicated asterisks (***P < 0.001; Dunnett’s test). 1.0 x 10^3^ conidia of the GPChBP (WT), GPChBΔ1–20 (Δ1–20), GPChBΔ21–40 (Δ21–40), GPChBΔ41–60 (Δ41–60), GPChBΔ61–80 (Δ61–80), GPChBΔ81–100 (Δ81–100), GPChBΔ100–115 (Δ100–115), and GPChBΔ115–140 (Δ115–140) strains were inoculated onto MMGpuu plates and incubated for 72 h at 37 °C (JPG 248 KB)

Supplementary file10 N-terminal disordered region is conserved in Class III chitin synthase. A Amino acid sequences of *A. fumigatus* ChsC (AfChsC), *A. fumigatus* ChsG (AfChsG), *E. dermatitidis* Chs3 (EdChs3), *N. crassa* Chs1 (NcChs1), and *C. neoformans* Chs7 (CnChs7) were analyzed by PrDOS server. B These amino acid sequences and the sequence of *A. nidulans* ChsB (AnChsB) were aligned by clustalW (https://www.genome.jp/tools-bin/clustalw), and N-terminal regions were shown. The orange box indicates a highly conserved region after the N-terminal disordered region. Amino acids 41–60 and 81–100 of AnChsB and their corresponding amino acids of other chitin synthases are indicated as cyan and magenta boxes, respectively (JPG 1816 KB)

Supplementary file11 (DOCX 30 KB)

Supplementary file12 (DOCX 27 KB)

## Data Availability

The datasets generated during and/or analyzed during the current study are available from the corresponding author on reasonable request.

## Abbreviations

CFW: Calcofluor white
CMAC: 7-Amino-4-chloromethylcoumarin
COPII: Coat protein complex II
EGFP: Enhanced green fluorescent protein
ER: Endoplasmic reticulum
SDS: Sodium dodecyl sulfate
PBS: Phosphate buffered saline

## References

[CR1] Albuquerque CP, Smolka MB, Payne SH (2008). A multidimensional chromatography technology for in-depth phosphoproteome analysis. Mol Cell Proteom.

[CR2] Blom N, Gammeltoft S, Brunak S (1999). Sequence and structure-based prediction of eukaryotic protein phosphorylation sites. J Mol Biol.

[CR3] Blom N, Sicheritz-Pontén T, Gupta R (2004). Prediction of post-translational glycosylation and phosphorylation of proteins from the amino acid sequence. Proteomics.

[CR4] Borgia PT, Iartchouk N, Riggle PJ (1996). The *chsB* gene of *Aspergillus nidulans* is necessary for normal hyphal growth and development. Fungal Genet Biol.

[CR5] Chen W, Cao P, Liu Y (2022). Structural basis for directional chitin biosynthesis. Nature.

[CR6] Chen-Wu JL, Zwicker J, Bowen AR, Robbins PW (1992). Expression of chitin synthase genes during yeast and hyphal growth phases of *Candida albicans*. Mol Microbiol.

[CR7] Chin CF, Bennett AM, Ma WK (2012). Dependence of Chs2 ER export on dephosphorylation by cytoplasmic Cdc14 ensures that septum formation follows mitosis. Mol Biol Cell.

[CR8] Choi WJ, Santos B, Durán A, Cabib E (1994). Are yeast chitin synthases regulated at the transcriptional or the posttranslational level?. Mol Cell Biol.

[CR9] Côte P, Hogues H, Whiteway M (2009). Transcriptional Analysis of the *Candida albicans* Cell Cycle. Mol Biol Cell.

[CR10] Doray B, Lee I, Knisely J (2007). The γ/σ1 and α/σ2 hemicomplexes of clathrin adaptors AP-1 and AP-2 harbor the dileucine recognition site. Mol Biol Cell.

[CR11] Dunker AK, Lawson JD, Brown CJ (2001). Intrinsically disordered protein. J Mol Graphics Model.

[CR12] Fajardo-Somera RA, Jöhnk B, Bayram Ö (2015). Dissecting the function of the different chitin synthases in vegetative growth and sexual development in *Neurospora crassa*. Fungal Genet Biol.

[CR13] Fernandes C, Gow NAR, Gonçalves T (2016). The importance of subclasses of chitin synthase enzymes with myosin-like domains for the fitness of fungi. Fungal Biol Rev.

[CR14] Feyder S, de Craene J-O, Bär S (2015). Membrane trafficking in the yeast *Saccharomyces cerevisiae* model. Int J Mol Sci.

[CR15] Free SJ (2013). Fungal cell wall organization and biosynthesis. Advances in genetics.

[CR16] Fukuda K, Yamada K, Deoka K (2009). Class III chitin synthase ChsB of *Aspergillus nidulans* localizes at the sites of polarized cell wall synthesis and is required for conidial development. Eukaryot Cell.

[CR17] García-Rodriguez LJ, Trilla JA, Castro C (2000). Characterization of the chitin biosynthesis process as a compensatory mechanism in the *fks1* mutant of *Saccharomyces cerevisiae*. FEBS Lett.

[CR18] Gohlke S, Muthukrishnan S, Merzendorfer H (2017). In vitro and in vivo studies on the structural organization of Chs3 from *Saccharomyces cerevisiae*. Int J Mol Sci.

[CR19] Gow NAR, Latge J-P, Munro CA (2017). The fungal cell wall: structure, biosynthesis, and function. Microbiol Spectr.

[CR20] Hernández-González M, Bravo-Plaza I, Pinar M (2018). Endocytic recycling via the TGN underlies the polarized hyphal mode of life. PLoS Genet.

[CR21] Horiuchi H (2009). Functional diversity of chitin synthases of *Aspergillus nidulans* in hyphal growth, conidiophore development and septum formation. Med Mycol.

[CR22] Ishida T, Kinoshita K (2007). PrDOS: prediction of disordered protein regions from amino acid sequence. Nucleic Acids Res.

[CR23] Jakobsen MK, Cheng Z, Lam SK (2013). Phosphorylation of Chs2p regulates interaction with COPII. J Cell Sci.

[CR24] Jin J, Iwama R, Takagi K, Horiuchi H (2021). AP-2 complex contributes to hyphal-tip-localization of a chitin synthase in the filamentous fungus *Aspergillus nidulans*. Fungal Biol.

[CR25] Jumper J, Evans R, Pritzel A (2021). Highly accurate protein structure prediction with AlphaFold. Nature.

[CR26] Katayama T, Uchida H, Ohta A, Horiuchi H (2012). Involvement of protein kinase C in the suppression of apoptosis and in polarity establishment in *Aspergillus nidulans* under conditions of heat stress. PLoS One.

[CR27] Kelly BT, McCoy AJ, Späte K (2008). A structural explanation for the binding of endocytic dileucine motifs by the AP2 complex. Nature.

[CR28] Köhler JR, Hube B, Puccia R, Casadevall A, Perfect JR (2017) Fungi that infect humans. Microbiol Spectr 5(3). 10.1128/microbiolspec.FUNK-0014-2016 10.1128/microbiolspec.funk-0014-2016 PMC11687496 28597822

[CR29] Lenardon MD, Milne SA, Mora-Montes HM (2010). Phosphorylation regulates polarisation of chitin synthesis in *Candida albicans*. J Cell Sci.

[CR30] Li X, Gerber SA, Rudner AD (2007). Large-scale phosphorylation analysis of α-factor-arrested *Saccharomyces cerevisiae*. J Proteome Res.

[CR31] Martínez-Rucobo FW, Eckhardt-Strelau L, Terwisscha Van Scheltinga AC (2009). Yeast chitin synthase 2 activity is modulated by proteolysis and phosphorylation. Biochem J.

[CR32] Merzendorfer H (2011). The cellular basis of chitin synthesis in fungi and insects: common principles and differences. Eur J Cell Biol.

[CR33] Munro CA, Schofield DA, Gooday GW, Gow NAR (1998). Regulation of chitin synthesis during dimorphic growth of *Candida albicans*. Microbiology.

[CR34] Munro CA, Selvaggini S, de Bruijn I (2007). The PKC, HOG and Ca^2+^ signalling pathways co-ordinately regulate chitin synthesis in *Candida albicans*. Mol Microbiol.

[CR35] Oh Y, Chang K-J, Orlean P (2012). Mitotic exit kinase Dbf2 directly phosphorylates chitin synthase Chs2 to regulate cytokinesis in budding yeast. Mol Biol Cell.

[CR36] Ohno H, Stewart J, Fournier M-C (1995). Interaction of tyrosine-based sorting signals with clathrin-associated proteins. Science.

[CR37] Owen DJ, Evans PR (1998). A structural explanation for the recognition of tyrosine-based endocytotic signals. Science.

[CR38] Pammer M, Briza P, Ellinger A (1992). *DIT101* (*CSD2*, *CAL1*), a cell cycle-regulated yeast gene required for synthesis of chitin in cell walls and chitosan in spore walls. Yeast.

[CR39] Ren Z, Chhetri A, Guan Z (2022). Structural basis for inhibition and regulation of a chitin synthase from *Candida albicans*. Nat Struct Mol Biol.

[CR40] Rogg LE, Fortwendel JR, Juvvadi PR, Steinbach WJ (2012). Regulation of expression, activity and localization of fungal chitin synthases. Med Mycol.

[CR41] Sacristan C, Manzano-Lopez J, Reyes A (2013). Oligomerization of the chitin synthase Chs3 is monitored at the Golgi and affects its endocytic recycling. Mol Microbiol.

[CR42] Sánchez N, Roncero C (2022). Chitin synthesis in yeast: a matter of trafficking. Int J Mol Sci.

[CR43] Shaw JA, Mol PC, Bowers B (1991). The function of chitin synthases 2 and 3 in the *Saccharomyces cerevisiae* cell cycle. J Cell Biol.

[CR44] Spellman PT, Sherlock G, Zhang MQ (1998). Comprehensive identification of cell cycle–regulated genes of the yeast *Saccharomyces cerevisiae* by microarray hybridization. Mol Biol Cell.

[CR45] Sudoh M, Nagahashi S, Doi M (1993). Cloning of the chitin synthase 3 gene from *Candida albicans* and its expression during yeast-hyphal transition. Mol Gen Genet.

[CR46] Swaney DL, Beltrao P, Starita L (2013). Global analysis of phosphorylation and ubiquitylation cross-talk in protein degradation. Nat Methods.

[CR47] Takeshita N, Ohta A, Horiuchi H (2002). *csmA*, a gene encoding a class V chitin synthase with a myosin motor-like domain of *Aspergillus nidulans*, is translated as a single polypeptide and regulated in response to osmotic conditions. Biochem Biophys Res Commun.

[CR48] Takeshita N, Yamashita S, Ohta A, Horiuchi H (2006). *Aspergillus nidulans* class V and VI chitin synthases CsmA and CsmB, each with a myosin motor-like domain, perform compensatory functions that are essential for hyphal tip growth. Mol Microbiol.

[CR49] Takeshita N, Wernet V, Tsuizaki M (2015). Transportation of *Aspergillus nidulans* class III and V chitin synthases to the hyphal tips depends on conventional kinesin. PLoS One.

[CR50] Teh EM, Chai CC, Yeong FM (2009). Retention of Chs2p in the ER requires N-terminal CDK1-phosphorylation sites. Cell Cycle.

[CR51] Tsuizaki M, Ohta A, Horiuchi H (2013). Myosin motor-like domain of class VI chitin synthase CsmB of *Aspergillus nidulans* is not functionally equivalent to that of class V chitin synthase CsmA. Biosci Biotechnol Biochem.

[CR52] Uversky VN (2019). Intrinsically disordered proteins and their “mysterious” (meta)physics. Front Phys.

[CR53] Valdivia RH, Schekman R (2003). The yeasts Rho1p and Pkc1p regulate the transport of chitin synthase III (Chs3p) from internal stores to the plasma membrane. Proc Natl Acad Sci USA.

[CR54] Weiskoff AM, Fromme JC (2014). Distinct N-terminal regions of the exomer secretory vesicle cargo Chs3 regulate its trafficking itinerary. Front Cell Dev Biol.

[CR55] Wright PE, Dyson HJ (2015). Intrinsically disordered proteins in cellular signalling and regulation. Nat Rev Mol Cell Biol.

[CR56] Yanai K, Kojima N, Takaya N (1994). Isolation and characterization of two chitin synthase genes from *Aspergillus nidulans*. Biosci Biotechnol Biochem.

[CR57] Zhou L, Evangelinos M, Wernet V (2018). Superresolution and pulse-chase imaging reveal the role of vesicle transport in polar growth of fungal cells. Sci Adv.

