# Supplementary figures and images for: The N-terminal disordered region of ChsB regulates its efficient transport to the hyphal apical surface in Aspergillus nidulans
Source: Curr Genet. 2023 Apr 18;69(2-3):175–88. doi: 10.1007/s00294-023-01267-1 (PMC10163080; doi:10.1007/s00294-023-01267-1)

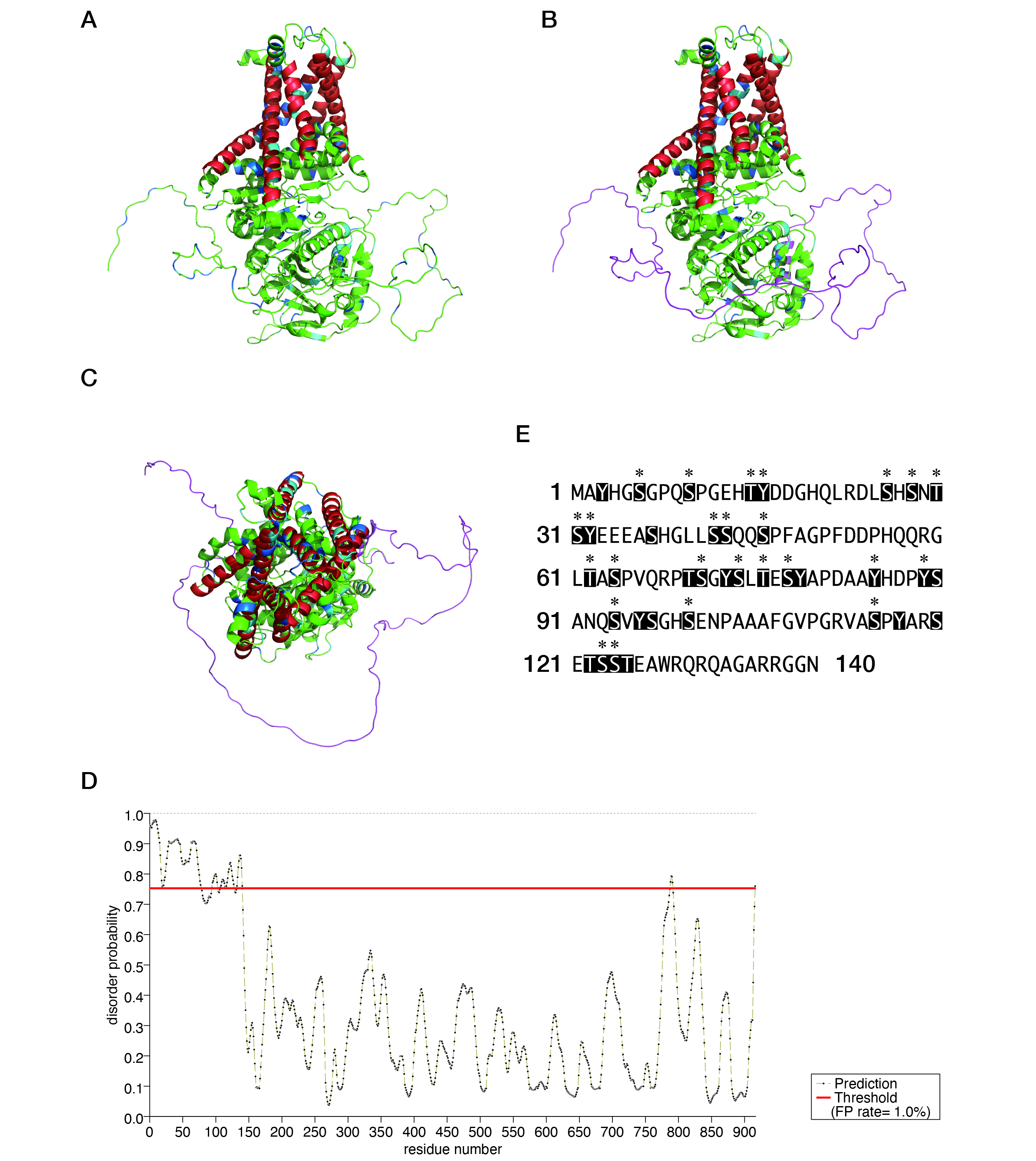

Supplement: Supplementary file 1 — Supplementary file1 ChsB is predicted to have an intrinsically disordered region at its N-terminus. A–D The predicted full-length structure of ChsB by AlphaFold2. A Side view of the structure. Red α-helices indicate predicted transmembrane domains. B The predicted disordered region at N-terminus is colored in magenta. C Top view of (B). D Amino acids sequences of ChsB were analyzed by the PrDOS server. E The first 140 amino acid sequences of ChsB are shown. Serine, threonine, and tyrosine residues are highlighted. Asterisks indicate predicted phosphorylation sites by NetPhos-3.1 (JPG 2522 KB) [file 294_2023_1267_MOESM1_ESM.jpg]

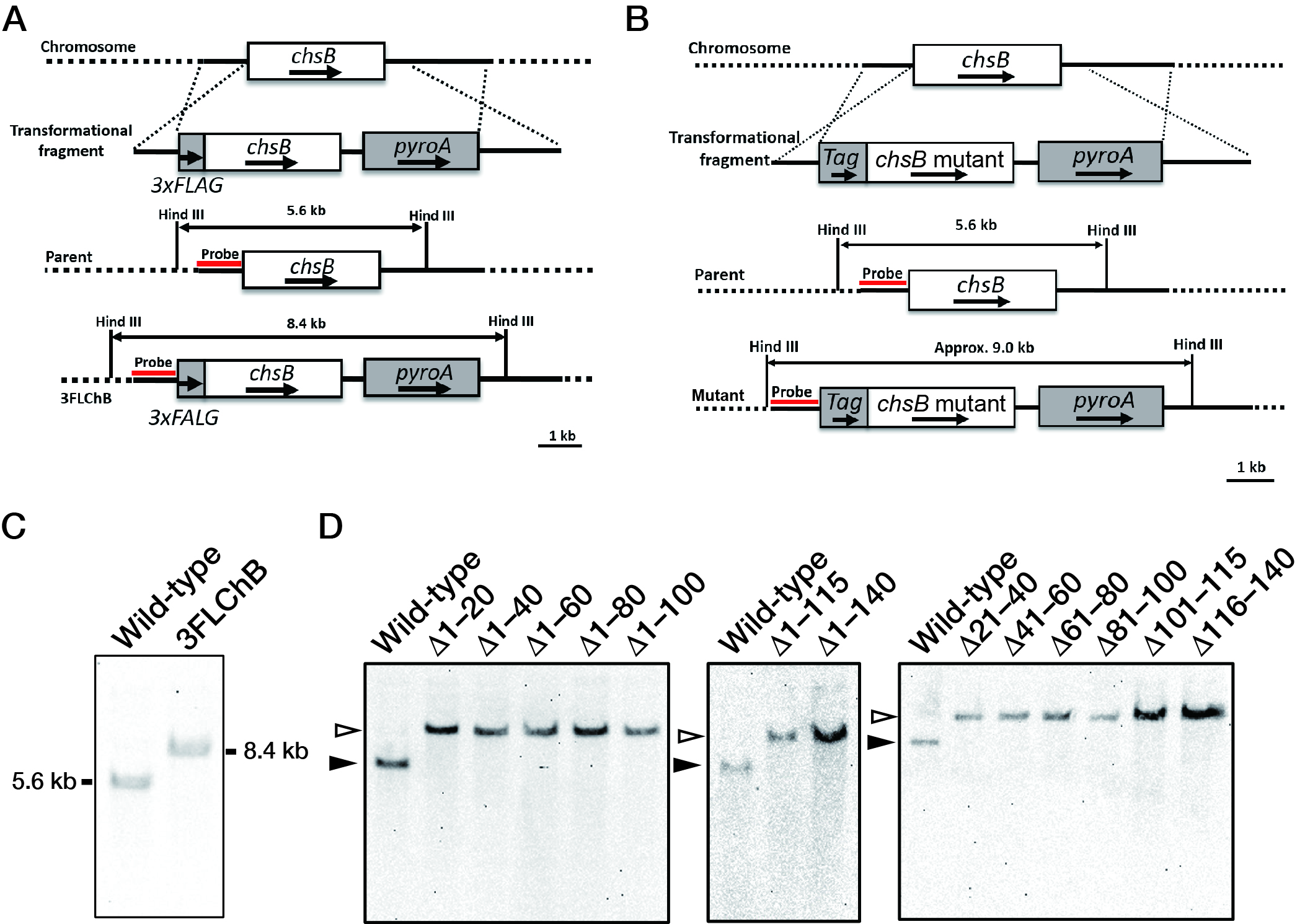

Supplement: Supplementary file 2 — Supplementary file2 Southern analyses of the strains constructed in this study. A, B Schemes of the southern analysis are depicted. C, D The results of the analysis. Black and white arrowheads indicate the postions at 5.4-kb and 9.0-kb bands, respectively. Panels A and B are the schemes of Panels C and D, respectively (JPG 1972 KB) [file 294_2023_1267_MOESM2_ESM.jpg]

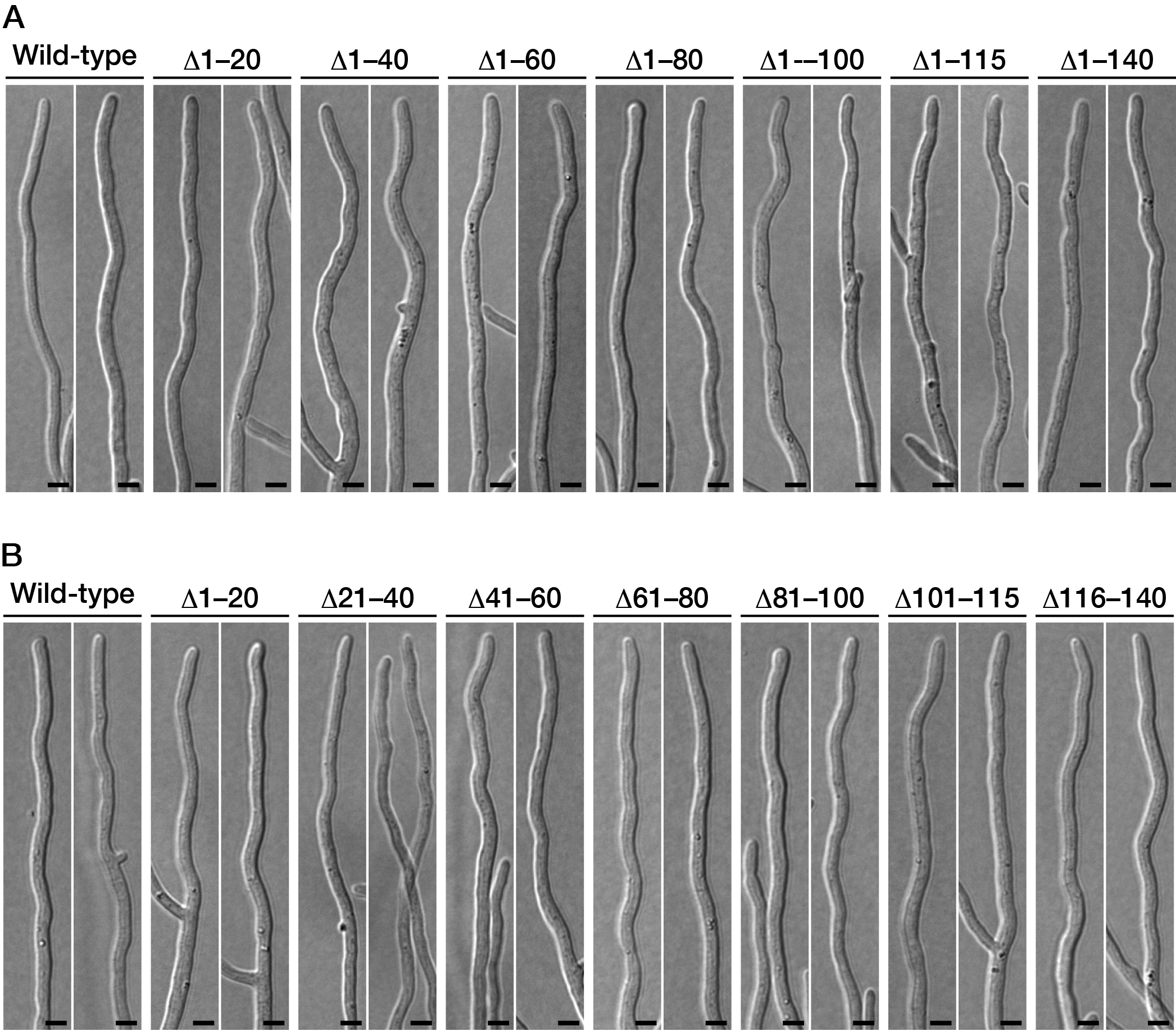

Supplement: Supplementary file 3 — Supplementary file3 Truncations or the amino acid deletions of the N-terminal disordered region did not affect hyphal morphology. A Conidia of the GPChBP (Wild-type), GPChBΔ1–20 (Δ1–20), GPChBΔ1–40 (Δ1–40), GPChBΔ1–60 (Δ1–60), GPChBΔ1–80 (Δ1–80), GPChBΔ1–100 (Δ1–100), GPChBΔ1–115 (Δ1–115), and GPChBΔ1–140 (Δ1–140) strains were inoculated onto MMGpuu plates and incubated for 22 h at 30 °C, after which the hyphal morphologies were observed under a microscope. B Conidia of the GPChBP (Wild-type), GPChBΔ1–20 (Δ1–20), GPChBΔ21–40 (Δ21–40), GPChBΔ41–60 (Δ41–60), GPChBΔ61–80 (Δ61–80), GPChBΔ81–100 (Δ81–100), GPChBΔ100–115 (Δ100–115), and GPChBΔ115–140 (Δ115–140) strains were inoculated onto MMGpuu plates and incubated for 22 h at 30 °C, after which the hyphal morphologies were observed under a microscope. Bars: 5 µm (JPG 1657 KB) [file 294_2023_1267_MOESM3_ESM.jpg]

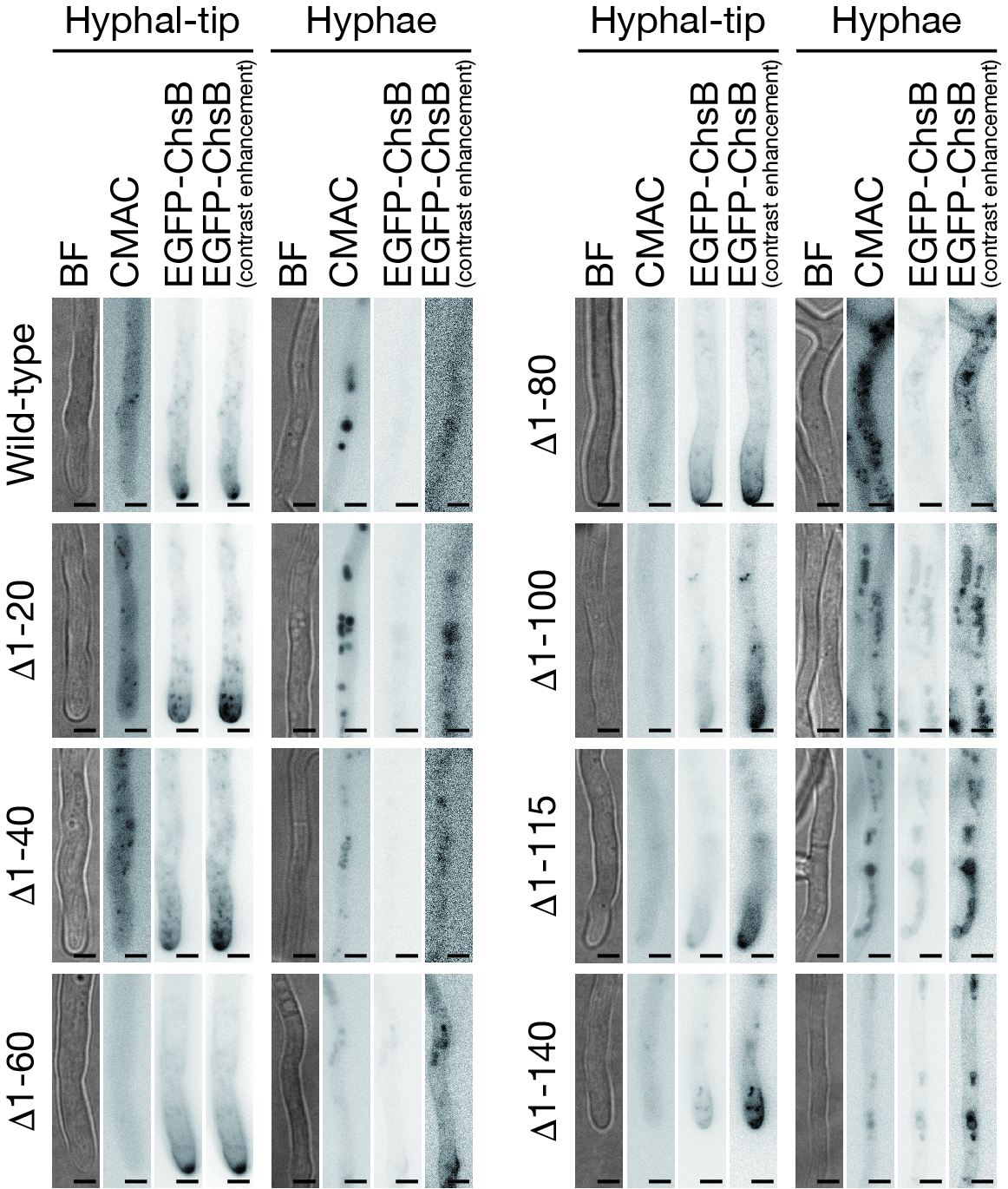

Supplement: Supplementary file 4 — Supplementary file4 Deletion of amino acids 1–100, 1-115, and 1-140 strongly alter ChsB localization to the vacuoles. Conidia of the GPChBP (Wild-type), GPChBΔ1–20 (Δ1–20), GPChBΔ1–40 (Δ1–40), GPChBΔ1–60 (Δ1–60), GPChBΔ1–80 (Δ1–80), GPChBΔ1–100 (Δ1–100), GPChBΔ1–115 (Δ1–115), and GPChBΔ1–140 (Δ1–140) strains were inoculated onto MMGpuu plates and incubated for 22 h at 30 °C, after which the hyphae were treated with CMAC-Ala-Pro and were observed under a fluorescence microscope. Bars: 3 µm (JPG 1817 KB) [file 294_2023_1267_MOESM4_ESM.jpg]

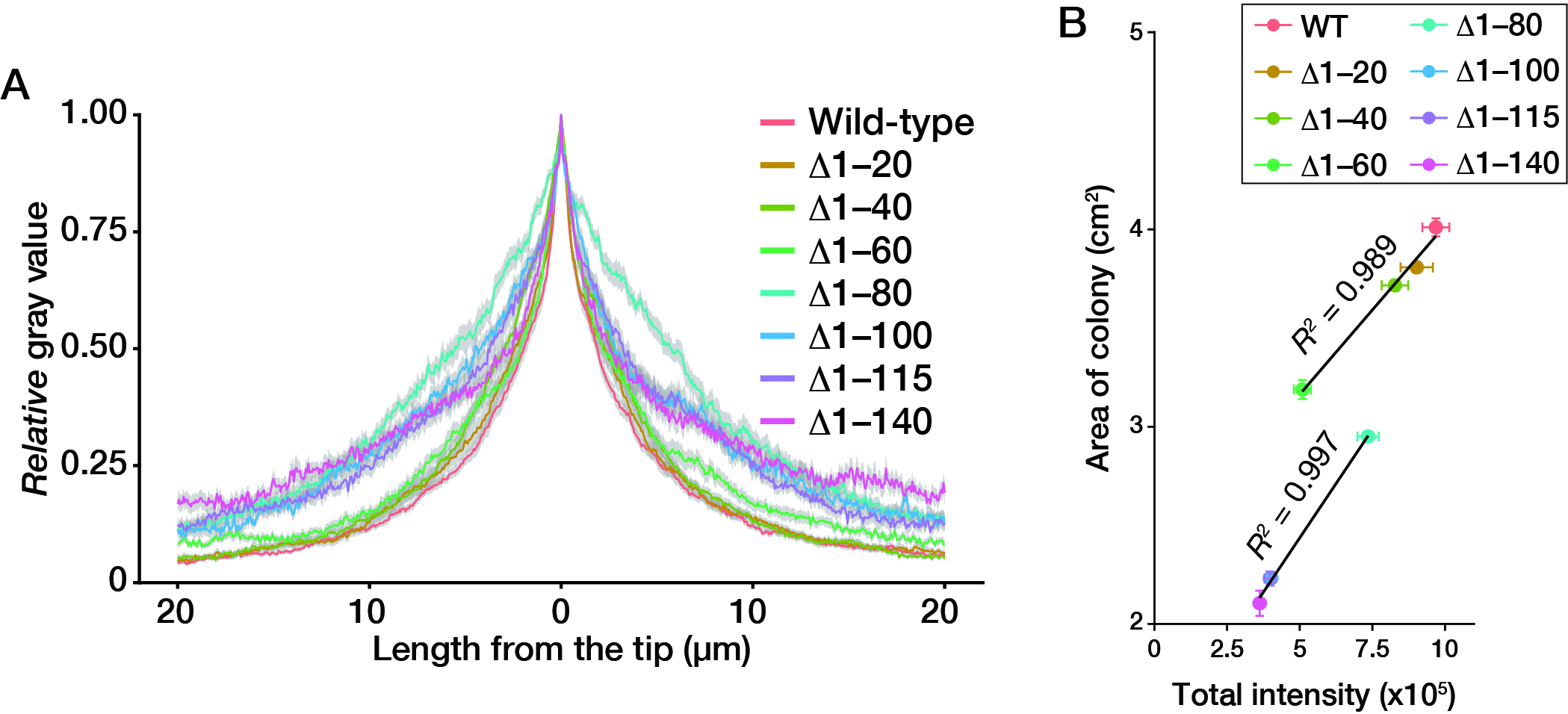

Supplement: Supplementary file 5 — Supplementary file5 Endocytosis of ChsB and total ChsB abundance on the hyphal surface are both involved in the hyphal elongation. A Using the data in Fig. 2B, relative values of the intensities were calculated concerning those at the hyphal tips. The intensities at the hyphal tips (0 μm) were set to 1. B The fluorescence intensities in Fig. 2D within 20 µm from the hyphal tips (0 μm) were calculated, and then the intensity and the area of the colony in Fig. 2B were plotted; dots indicate the mean and error bars represent S.E. Linear regression analysis was performed in each group (JPG 883 KB) [file 294_2023_1267_MOESM5_ESM.jpg]

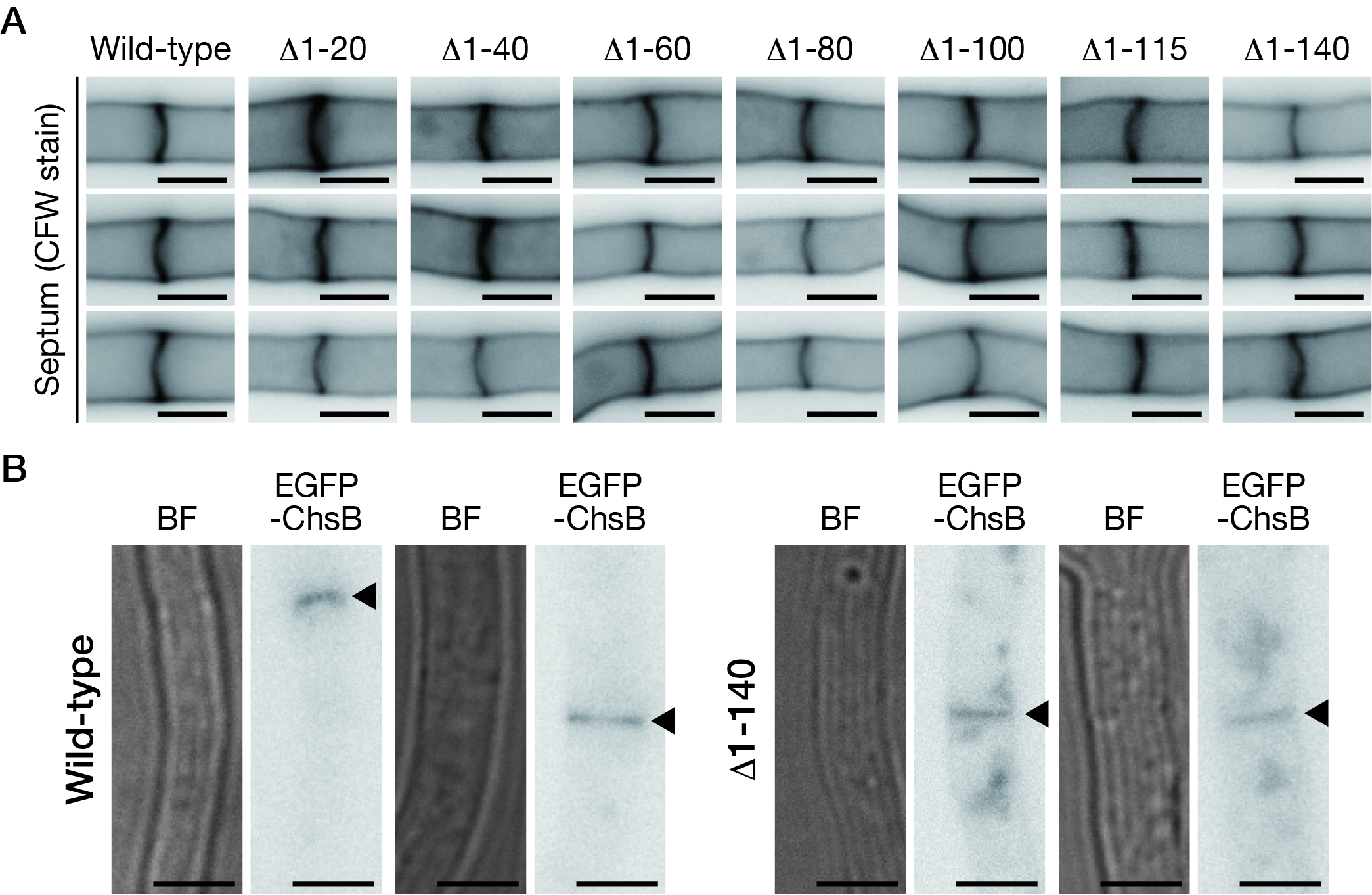

Supplement: Supplementary file 6 — Supplementary file6 Truncations of the N-terminal disordered region did not affect septum formation. A Conidia of the GPChBP (Wild-type), GPChBΔ1–20 (Δ1–20), GPChBΔ1–40 (Δ1–40), GPChBΔ1–60 (Δ1–60), GPChBΔ1–80 (Δ1–80), GPChBΔ1–100 (Δ1–100), GPChBΔ1–115 (Δ1–115), and GPChBΔ1–140 (Δ1–140) strains were inoculated onto MMGpuu plates and incubated for 20 h at 30 °C, after which the hyphae were treated with CFW and were observed under a fluorescence microscope. B Conidia of the GPChBP and GPChBΔ1–140 strains were inoculated onto MMGpuu plates and incubated for 20 h at 30 °C, after which EGFP-ChsBs at forming septa were observed under a fluorescence microscope. Bars: 3 µm (JPG 1925 KB) [file 294_2023_1267_MOESM6_ESM.jpg]

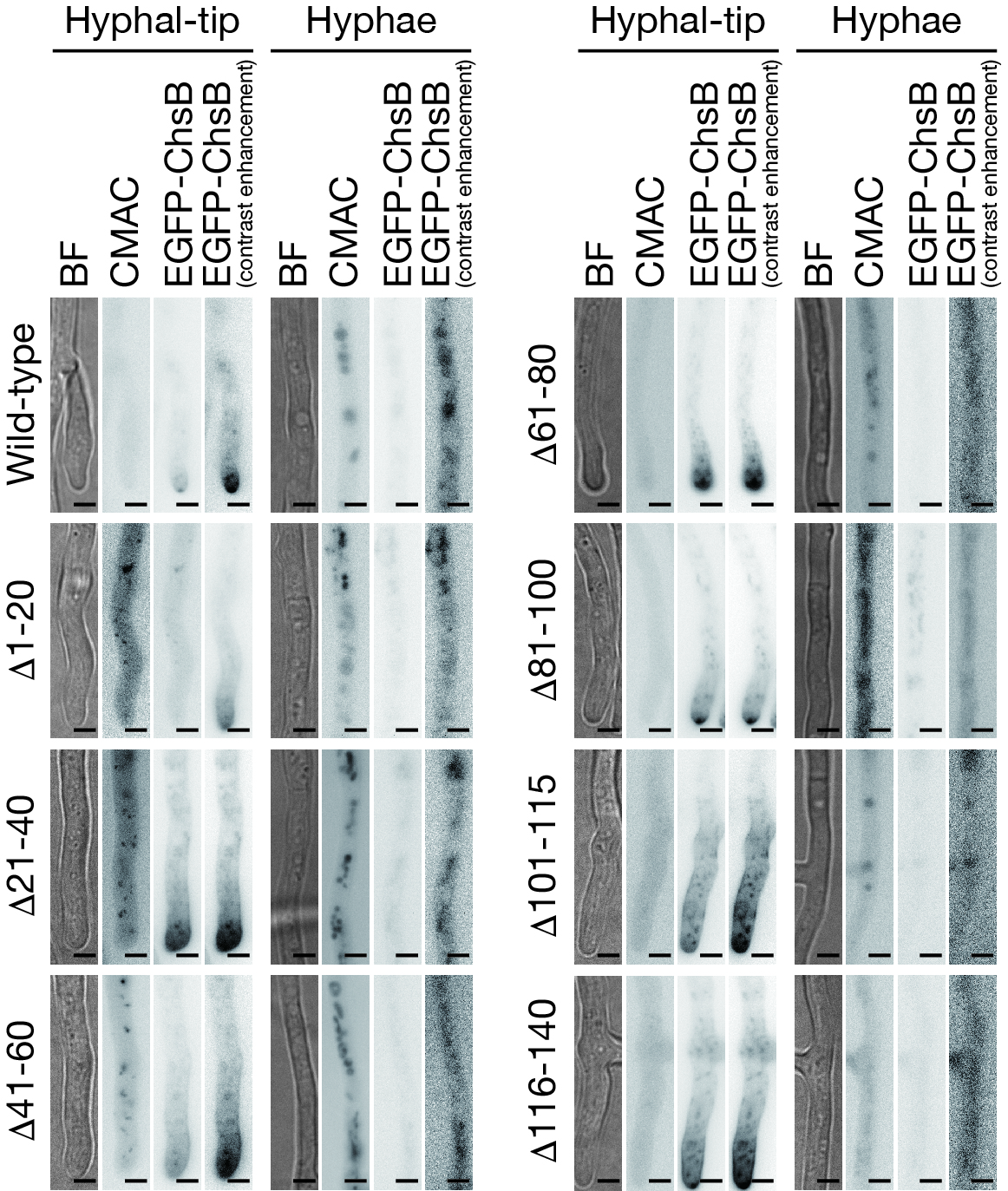

Supplement: Supplementary file 7 — Supplementary file7 None of the 20-amino acid deletions of the N-terminal disordered region strengthened the vacuolar localization of ChsB. Conidia of the GPChBP (Wild-type), GPChBΔ1–20 (Δ1–20), GPChBΔ21–40 (Δ21–40), GPChBΔ41–60 (Δ41–60), GPChBΔ61–80 (Δ61–80), GPChBΔ81–100 (Δ81–100), GPChBΔ100–115 (Δ100–115), and GPChBΔ115–140 (Δ115–140) strains were inoculated onto MMGpuu plates and incubated for 20 h at 30 °C, after which the hyphae were treated with CMAC-Ala-Pro and were observed under a fluorescence microscope. Bars: 3 µm (JPG 1859 KB) [file 294_2023_1267_MOESM7_ESM.jpg]

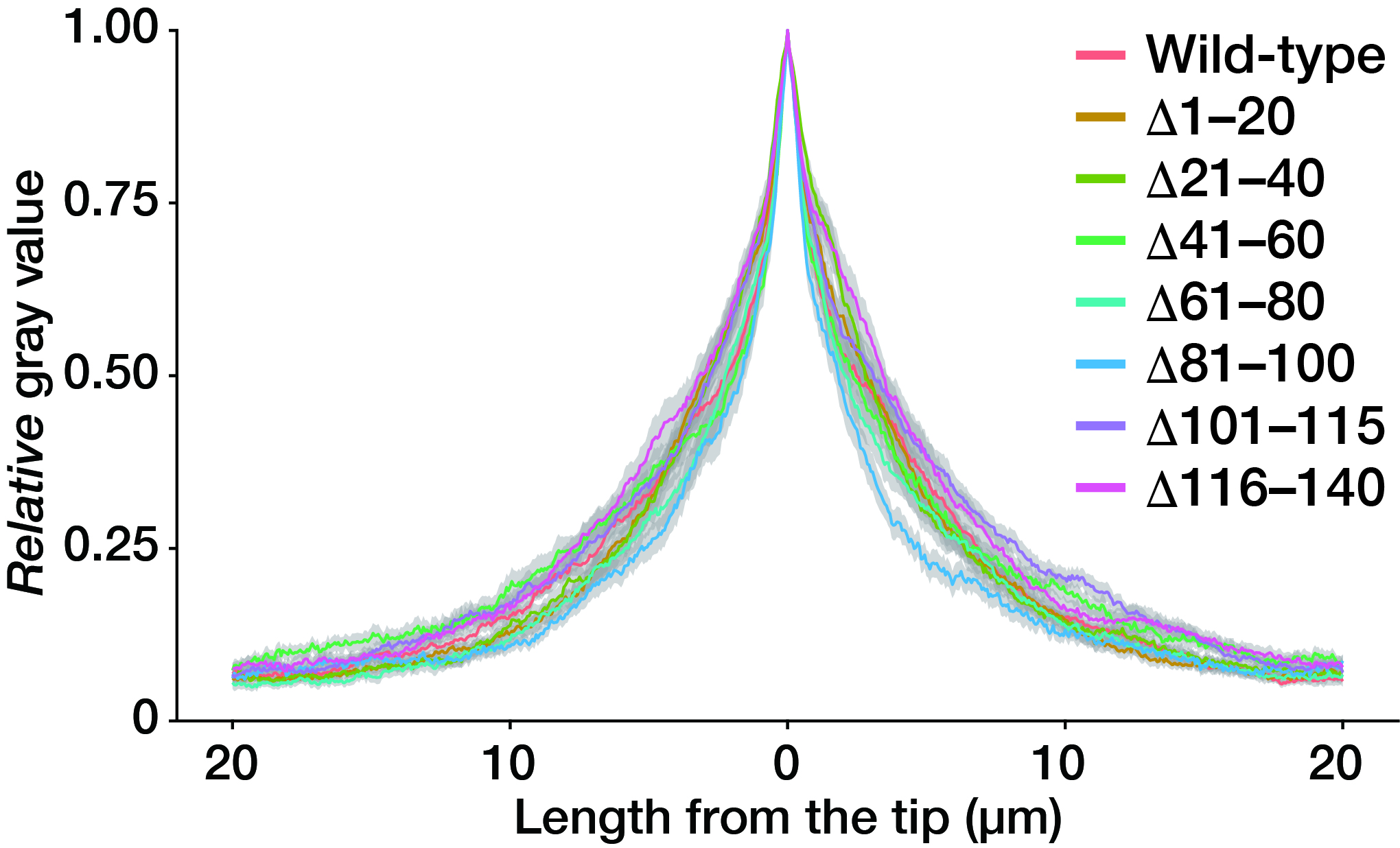

Supplement: Supplementary file 8 — Supplementary file8 None of the 20-amino acid deletions of ChsB affected the internalization rate of ChsB. Using the data in Fig. 4B, relative values of the intensities were calculated concerning those at the hyphal tips. The intensities at the hyphal tips (0 μm) were set to 1 (JPG 974 KB) [file 294_2023_1267_MOESM8_ESM.jpg]

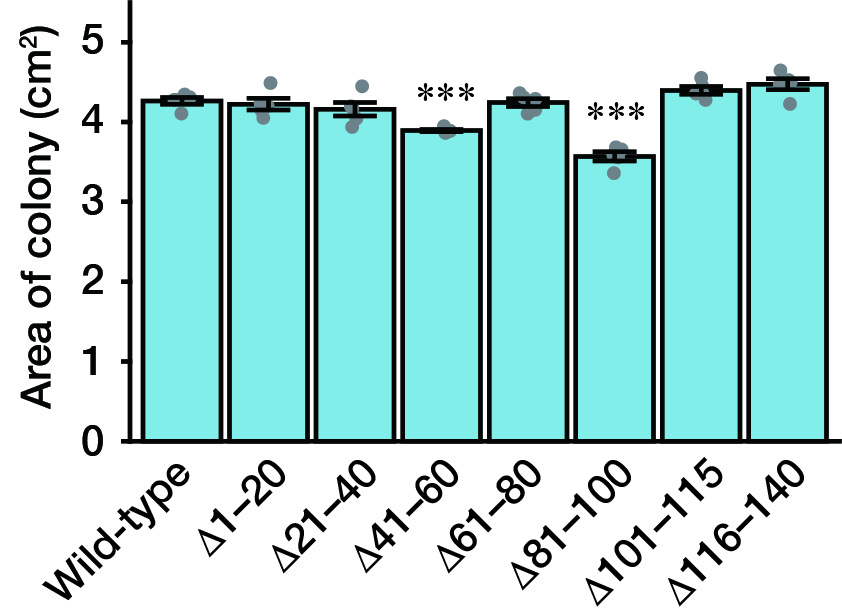

Supplement: Supplementary file 9 — Supplementary file9 Deletion of amino acids 41–60 retarded the growth at a high temperature. Colony areas of the strains described below were measured. Bars indicate the mean of five independent experiments, and dots indicate raw data. Error bars represent S.E. Significant differences compared to the wild-type are indicated asterisks (***P < 0.001; Dunnett’s test). 1.0 x 10^3^ conidia of the GPChBP (WT), GPChBΔ1–20 (Δ1–20), GPChBΔ21–40 (Δ21–40), GPChBΔ41–60 (Δ41–60), GPChBΔ61–80 (Δ61–80), GPChBΔ81–100 (Δ81–100), GPChBΔ100–115 (Δ100–115), and GPChBΔ115–140 (Δ115–140) strains were inoculated onto MMGpuu plates and incubated for 72 h at 37 °C (JPG 248 KB) [file 294_2023_1267_MOESM9_ESM.jpg]

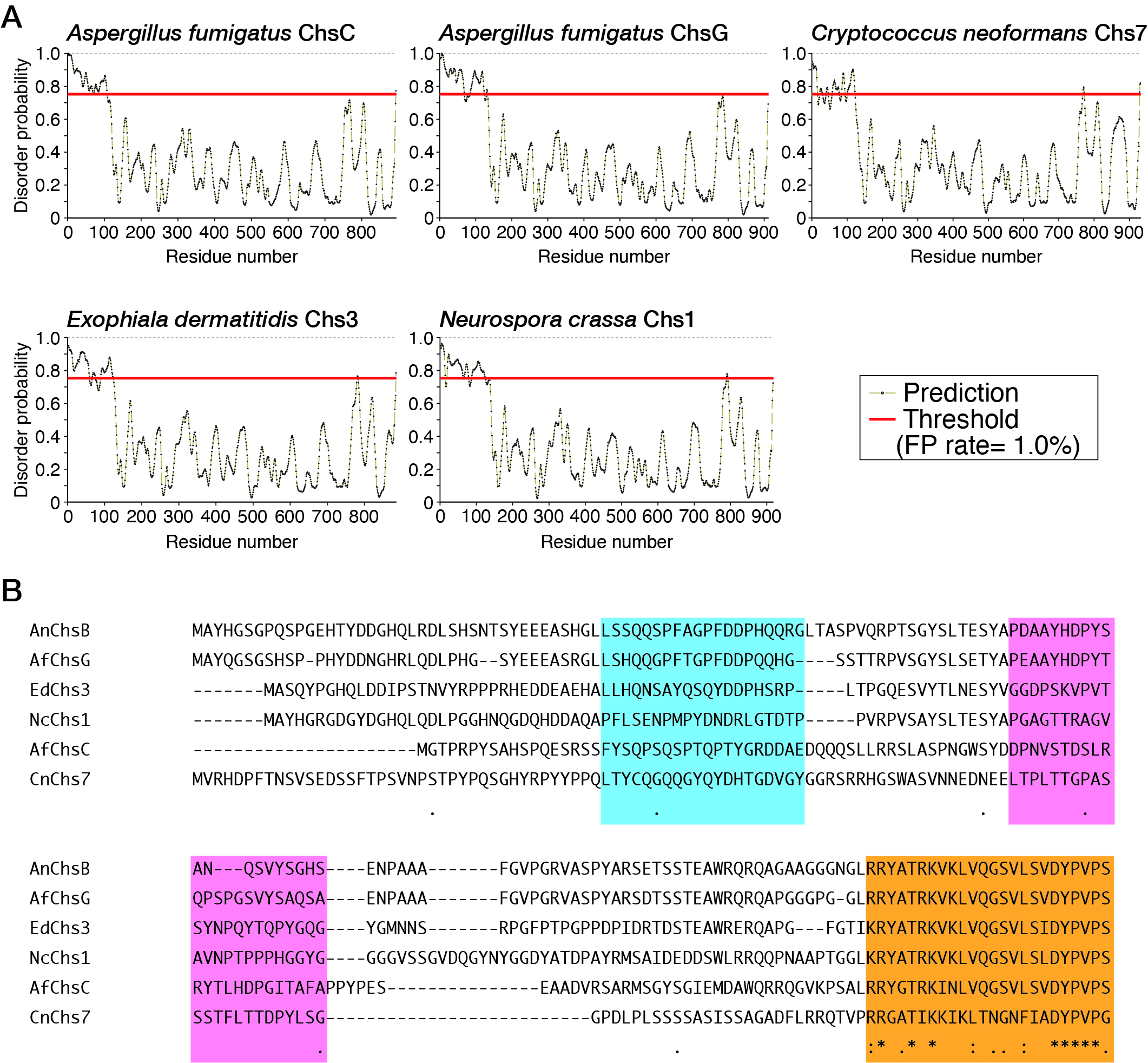

Supplement: Supplementary file 10 — Supplementary file10 N-terminal disordered region is conserved in Class III chitin synthase. A Amino acid sequences of A. fumigatus ChsC (AfChsC), A. fumigatus ChsG (AfChsG), E. dermatitidis Chs3 (EdChs3), N. crassa Chs1 (NcChs1), and C. neoformans Chs7 (CnChs7) were analyzed by PrDOS server. B These amino acid sequences and the sequence of A. nidulans ChsB (AnChsB) were aligned by clustalW (https://www.genome.jp/tools-bin/clustalw), and N-terminal regions were shown. The orange box indicates a highly conserved region after the N-terminal disordered region. Amino acids 41–60 and 81–100 of AnChsB and their corresponding amino acids of other chitin synthases are indicated as cyan and magenta boxes, respectively (JPG 1816 KB) [file 294_2023_1267_MOESM10_ESM.jpg]
