# Supplementary material for: The N-terminal disordered region of ChsB regulates its efficient transport to the hyphal apical surface in Aspergillus nidulans
Source: Curr Genet. 2023 Apr 18;69(2-3):175–88. doi: 10.1007/s00294-023-01267-1 (PMC10163080; doi:10.1007/s00294-023-01267-1)
Supplement: Supplementary file 11 — Supplementary file11 (DOCX 30 KB) [file 294_2023_1267_MOESM11_ESM.docx]

Table S1. Strains used in this study

Strain Genotype Source

A1149 *pyrG89 pyroA4 nkuA*::*argB* FGSC*

A1149/pyrG-1 *pyrG89*::*pyrG* *pyroA4* *nkuA*::*argB* Katayama et al., 2012

A26 *biA1* FGSC*

3FLChB *pyrG89 pyroA4 nkuA*::*argB* This study

*chsB*::*pyroA–3xflag–chsB*

3FLChBP *pyrG89*::*pyrG pyroA4 nkuA*::*argB* This study

*chsB*::*pyroA–3xflag–chsB*

GPChBP *pyrG89*::*pyrG pyroA4 nkuA*::*argB* Jin *et al.*, 2021

*chsB*::*pyroA–egfp–chsB*

GPChBPΔ1–20 *pyrG89*::*pyrG pyroA4 nkuA*::*argB* This study

*chsB*::*pyroA–egfp–chsB^Δ1–20^*

GPChBPΔ1–40 *pyrG89*::*pyrG pyroA4 nkuA*::*argB* This study

*chsB*::*pyroA–egfp–chsB^Δ1–40^*

GPChBPΔ1–60 *pyrG89*::*pyrG pyroA4 nkuA*::*argB* This study

*chsB*::*pyroA–egfp–chsB^Δ1–60^*

GPChBPΔ1–80 *pyrG89*::*pyrG pyroA4 nkuA*::*argB* This study

*chsB*::*pyroA–egfp–chsB^Δ1–80^*

GPChBPΔ1–100 *pyrG89*::*pyrG pyroA4 nkuA*::*argB* This study

*chsB*::*pyroA–egfp–chsB^Δ1–100^*

GPChBPΔ1–115 *pyrG89*::*pyrG pyroA4 nkuA*::*argB* This study

*chsB*::*pyroA–egfp–chsB^Δ1–115^*

GPChBPΔ1–140 *pyrG89*::*pyrG pyroA4 nkuA*::*argB* This study

*chsB*::*pyroA–egfp–chsB^Δ1–140^*

GPChBPΔ21–40 *pyrG89*::*pyrG pyroA4 nkuA*::*argB* This study

*chsB*::*pyroA–egfp–chsB^Δ21–40^*

GPChBPΔ41–60 *pyrG89*::*pyrG pyroA4 nkuA*::*argB* This study

*chsB*::*pyroA–egfp–chsB^Δ41–60^*

GPChBPΔ61–80 *pyrG89*::*pyrG pyroA4 nkuA*::*argB* This study

*chsB*::*pyroA–egfp–chsB^Δ61–80^*

GPChBPΔ81–100 *pyrG89*::*pyrG pyroA4 nkuA*::*argB* This study

*chsB*::*pyroA–egfp–chsB^Δ81–100^*

GPChBPΔ101–115 *pyrG89*::*pyrG pyroA4 nkuA*::*argB* This study

*chsB*::*pyroA–egfp–chsB^Δ101–115^*

GPChBPΔ116–140 *pyrG89*::*pyrG pyroA4 nkuA*::*argB* This study

*chsB*::*pyroA–egfp–chsB^Δ116–140^*

*Fungal Genetics Stock Center: Kansas State University, Manhattan, KS, USA
