# Supplementary material for: The N-terminal disordered region of ChsB regulates its efficient transport to the hyphal apical surface in Aspergillus nidulans
Source: Curr Genet. 2023 Apr 18;69(2-3):175–88. doi: 10.1007/s00294-023-01267-1 (PMC10163080; doi:10.1007/s00294-023-01267-1)
Supplement: Supplementary file 12 — Supplementary file12 (DOCX 27 KB) [file 294_2023_1267_MOESM12_ESM.docx]

Table S2. Primers used in this study

Name Sequence (5´→3´)

5'-YFP-chsB for CACACTACTATGGGAAACACCGAAGC

3xFLAG-chsB-1rev CTTGTCATCGTCATCCTTGTAATCGATGTCATGATCTTTATAATCAC

CGTCATGGTCTTTGTAGTCCATGGTTAAACTGGTAGTATGTGCGAA

TAGACG

3xFLAG-chsB-2 for ATGGACTACAAAGACCATGACGGTGATTATAAAGATCATGACATCG

ATTACAAGGATGACGATGACAAGATGGCCTACCACGGCTCTG

3'-YFP-chsB rev ATATCGTGAATCAGGCTACCACCTCC

pyrG ORF-1kb for CCGTCTTCCGAGAACGTCA

pyrG ORF rev TCAAAGTCCAACTCTTTTCTCGTAAGC

5'-9xHA-chsB for GGAAGCTTGGTTTTGTCGGAAGTGCAGC

3'-9xHA-chsB rev TCGGCTCAAGGAAAAGGAACTGC

3'-egfp rev GGACTTGTACAGCTCGTCCATGC

egfp-chsB-F AGCTTCACAATATACCAATGAGTGTGCACTCCAA

egfp-chsB-R TCCCAAGCAGCCAAGCGCC

egfp-chsB-Δ1-20-2-F GCATGGACGAGCTGTACAAGTCCCAGCTCCGCGACCTCTCAC

egfp-chsB-Δ1-40-2-F GCATGGACGAGCTGTACAAGTCCTTATCCAGCCAACAAAGCCCTT

TCG

egfp-chsB-Δ1-60-2-F GCATGGACGAGCTGTACAAGTCCCTTACCGCTTCACCCGTACAGC

egfp-chsB-Δ1-80-2-F GCATGGACGAGCTGTACAAGTCCCCCGACGCCGCATACCAT

egfp-chsB-Δ1-100-2-F GCATGGACGAGCTGTACAAGTCCGAGAACCCTGCAGCCGCTTTT

egfp-chsB-Δ1-115-F CCTTACGCTCGTAGTGAAACTTCGT

egfp-chsB-Δ1-140-F GGGCTTCGTCGTTATGCCACA

egfp-chsBΔ21-40-1-R ATGGCCGTCATCATAAGTATGCTCG

egfp-chsBΔ41-60-1-R CAATCCATGAGAGGCTTCTTCTTCGTAC

egfp-chsBΔ61-80-1-R GCCACGCTGCTGATGGG

egfp-chsBΔ81-100-1-R AGCGTAAGACTCAGTCAAACTGTATCC

egfp-chsBΔ101-115-1-R TGAGTGGCCGGAGTAGACCG

egfp-chsBΔ116-140-1-R CGATGCTACACGTCCAGGAACG

egfp-chsB-Δ21-40-2-F CGAGCATACTTATGATGACGGCCATTTATCCAGCCAACAAAGCCCT

TTCG

egfp-chsB-Δ41-60-2-F GTACGAAGAAGAAGCCTCTCATGGATTGCTTACCGCTTCACCCGT

ACAGC

egfp-chsB-Δ61-80-2-F CCCATCAGCAGCGTGGCCCCGACGCCGCATACCAT

egfp-chsB-Δ81-100-2-F GGATACAGTTTGACTGAGTCTTACGCTGAGAACCCTGCAGCCGCT

TT

egfp-chsB-Δ101-115-2-F CGGTCTACTCCGGCCACTCACCTTACGCTCGTAGTGAAACTTCGT

egfp-chsB-Δ116-140-2-F CGTTCCTGGACGTGTAGCATCGGGGCTTCGTCGTTATGCCACA
